# Supplementary material for: Evolutionary Dynamics of the OR Gene Repertoire in Teleost Fishes: Evidence of an Association with Changes in Olfactory Epithelium Shape
Source: Mol Biol Evol. 2021 May 5;38(9):3742–53. doi: 10.1093/molbev/msab145 (PMC8661438; doi:10.1093/molbev/msab145)
Supplement: msab145_Supplementary_Data [file msab145_supplementary_data.zip › Supplementary_Figures.pdf]

Supplementary Figures

Fig. S1. Congruence of genome size estimates. (A) Correlation between expected genome size (C-value x 0.978 x 10^9) and the assembly genome size. (B) Species with incongruent genome assembly size relative to their expected genome size.

Fig. S2. Correlation between the number of different types of OR genes. (A) Number of truncated genes *vs* number of pseudogenes. (B) Number of edge genes *vs* number of pseudogenes. (C) Number of edge genes *vs* number of truncated genes. (D) Number of functional genes *vs* proportion of pseudogenes.

Fig. S3. Correlation between the number of functional OR genes and the number of pseudogenes. (A) Ordinary least squares regression. (B) Phylogenetic generalized least squares regression.

Fig. S4. Comparison of OR gene repertoires from present and previous studies. (A) *Danio rerio* (Niimura 2009). (B) *Gasterosteus aculeatus* (Niimura 2009). (C) *Dichotomyctere nigroviridis* (Niimura 2009). (D) *Oryzias latipes* (Niimura 2009). (E) *Takifugu rubripes* (Niimura 2009). (F) *Ictalurus punctatus* (Gao et al. 2017). (G) *Pseudoliparis swirei* (Jiang et al. 2019). (H) *Siniperca chuatsi* (Lv et al. 2019).

Fig. S5. Phylogeny of functional OR genes retrieved from 38 teleost species across 38 orders. Branches are colored according to the gene family classification. Number and percentage of sequences belonging to each family are reported on the tree.

Fig. S6. Distribution of the number of functional OR genes in 163 teleost species.

Fig. S7. Birth and death rates reported along the branches of the chronogram in **fig. 2**.

Fig. S8. Correlation between number of gene losses inferred with NOTUNG and number of pseudogenes plus truncated genes found in external branches of the phylogenetic tree. Using only branches shorter than 5 Myr (A), 20 Myr (B), 40 Myr (C), no threshold (D).

Fig. S9. Variation in the correlation between the number of gene losses inferred with NOTUNG and the number of (A) pseudogenes, (B) truncated genes and (C) edge genes in external branches of the phylogenetic tree in **fig. 2** according to a maximum branch length threshold, from 2 to 200 Myr. For each threshold, the number of branches is indicated by a gray triangle.

Fig. S10. Pseudogene fade away. (A) Distribution of the number of generations after which a neutrally evolving OR coding sequence is not recognized by a tblastn with an e-value of 1e-20. The distribution results from 10,000 simulations. (B) Cumulative distribution.

Fig. S11. Chronogram, based on fishtreeoflife.org, of teleost species for which information on the olfactory epithelium shape is available in literature (ML: multi-lamellar, Non-ML: non multi-lamellar). The olfactory epithelium shapes for internal branches were inferred with PastML using: (A) Maximum likelihood MPPA with F81 model, (B) DOWNPASS parsimony, (C) DELTRAN parsimony, (D) ACCTRAN parsimony. Internal branches corresponding to the two highest OR death rates (see also **fig. 2**) are indicated by ovals. (E)

Chronogram of Tetraodontiformes based on fishtreeoflife.org for which information on the olfactory epithelium shape is available in literature. Ancestral states inferred by maximum likelihood or parsimony methods produced the same results. (F, G, H) Cladogram of Tetraodontiformes, based on family relationships in Arcila and Tyler (2017) and olfactory epithelium data from Tyler (1980). (F) Maximum likelihood MPPA with F81 model. (G) DOWNPASS parsimony. (H) DELTAN parsimony. (I) ACCTRAN parsimony. A star indicates a species which is not present in the molecular chronogram in (F, G, H, I).

Fig. S12. Correlations between olfactory epithelium shape and number of functional OR genes. (A) Phylogenetic logistic regression using data from species for which olfactory epithelium shape is known and species for which olfactory epithelium shape was inferred using data on species from the same genus. (B) Only species for which olfactory epithelium shape is known.

Fig. S13. Correlations between olfactory epithelium shape and number of pseudogenes. (A) Phylogenetic logistic regression between olfactory epithelium shape (1 for ML, 0 for non-ML) and number of pseudogenes. (B) Phylogenetic generalized least squares regression between the number of pseudogenes and number of lamellae in the olfactory epithelium.

Fig. S14. Correlation between number of functional OR genes and some morphological, ecological and genomic characteristics. (A) Maximum length. (B) Relative eye size (eye diameter/standard length). (C) Maximum depth range. (D) Preferred temperature. (E) Trophic level. (F) Genome size.

A

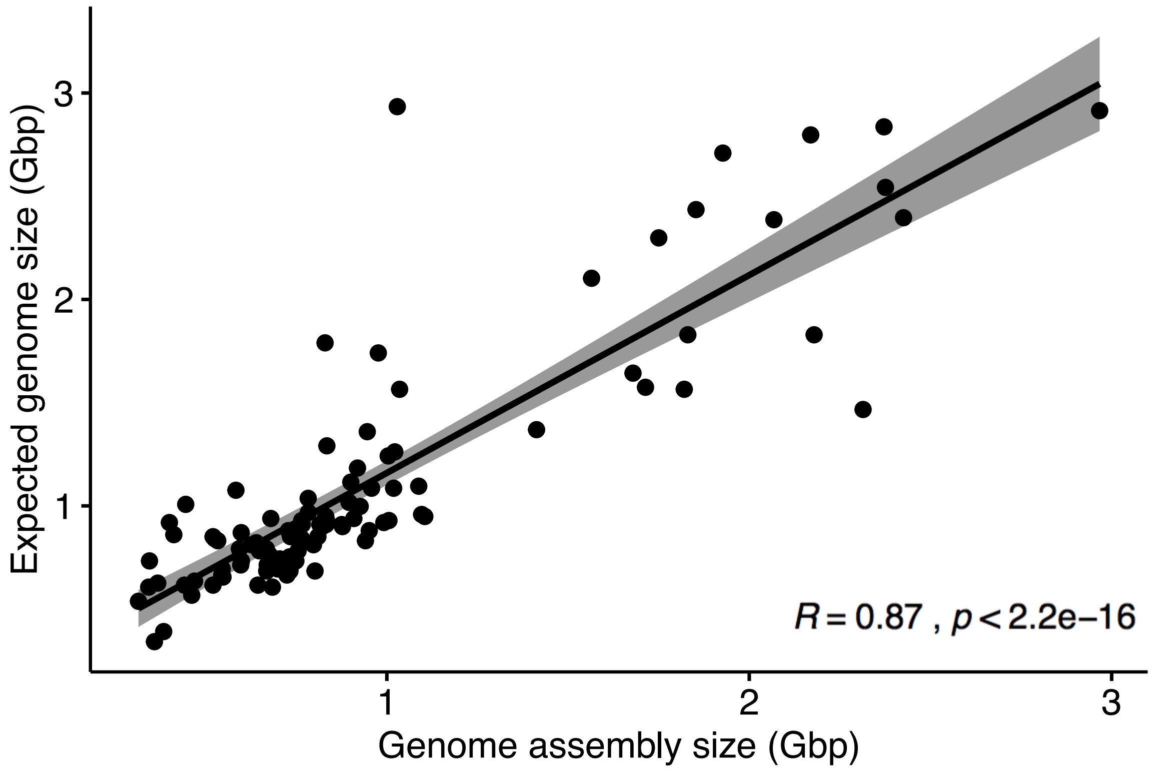

B

Species	Assembly size (bp)	Expected size (bp)	Difference (bp)
Chionodraco hamatus	829,466,164	1,789,740,000	-960,273,836
Colossoma macropomum	2,314,107,020	1,467,000,000	847,107,020
Nerophis ophidion	976,361,201	1,740,840,000	-764,478,799
Oncorhynchus nerka	1,927,125,257	2,709,060,000	-781,934,743
Opsanus beta	1,028,783,780	2,934,000,000	-1,905,216,220
Salvelinus alpinus	2,169,536,488	2,797,080,000	-627,543,512

Fig. S1

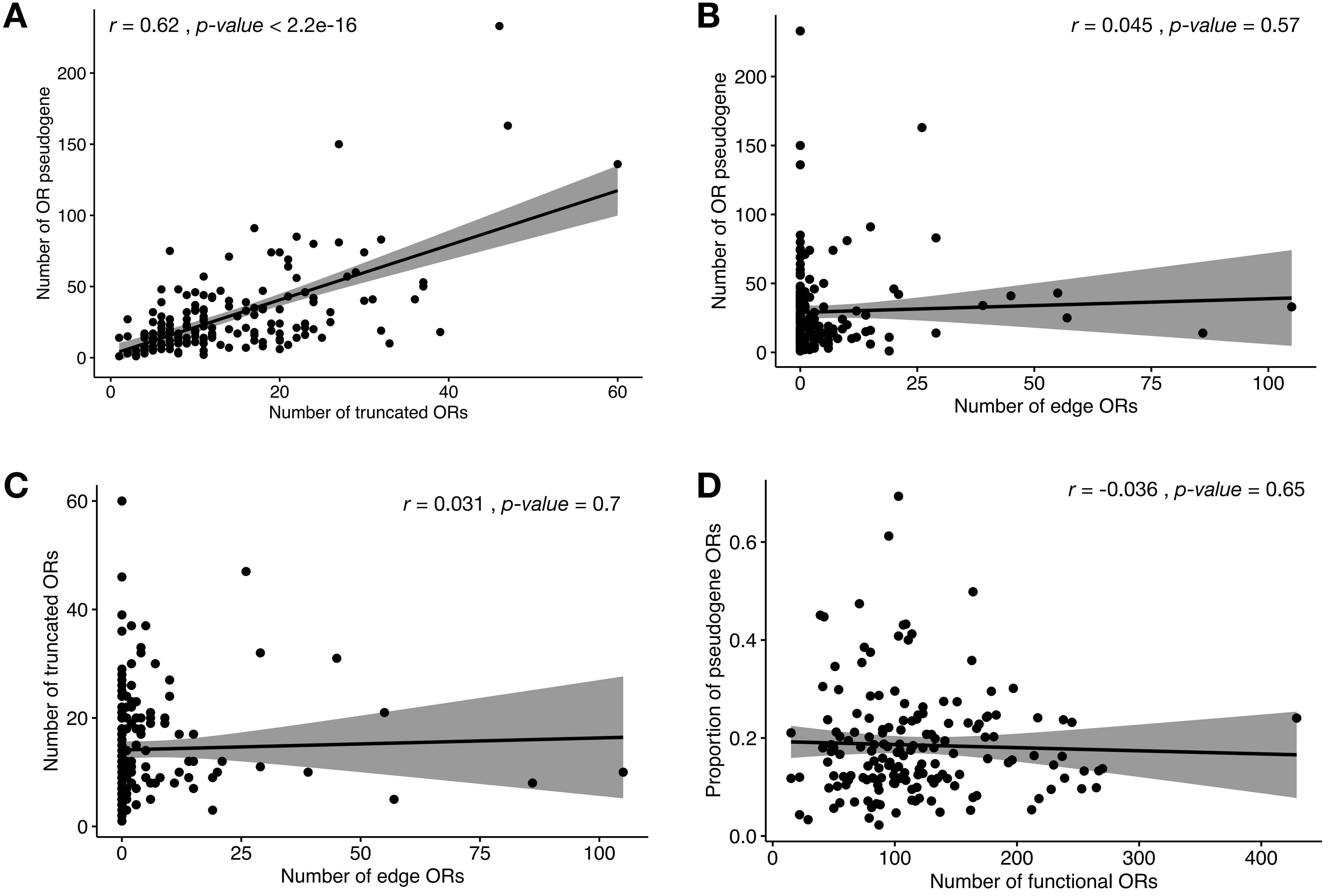
A
r = 0.62 , p-value < 2.2e-16
Number of OR pseudogene
Number of truncated ORs
B
r = 0.045 , p-value = 0.57
Number of OR pseudogene
Number of edge ORs
C
r = 0.031 , p-value = 0.7
Number of truncated ORs
Number of edge ORs
D
r = -0.036 , p-value = 0.65
Proportion of pseudogene ORs
Number of functional ORs

Fig. S2

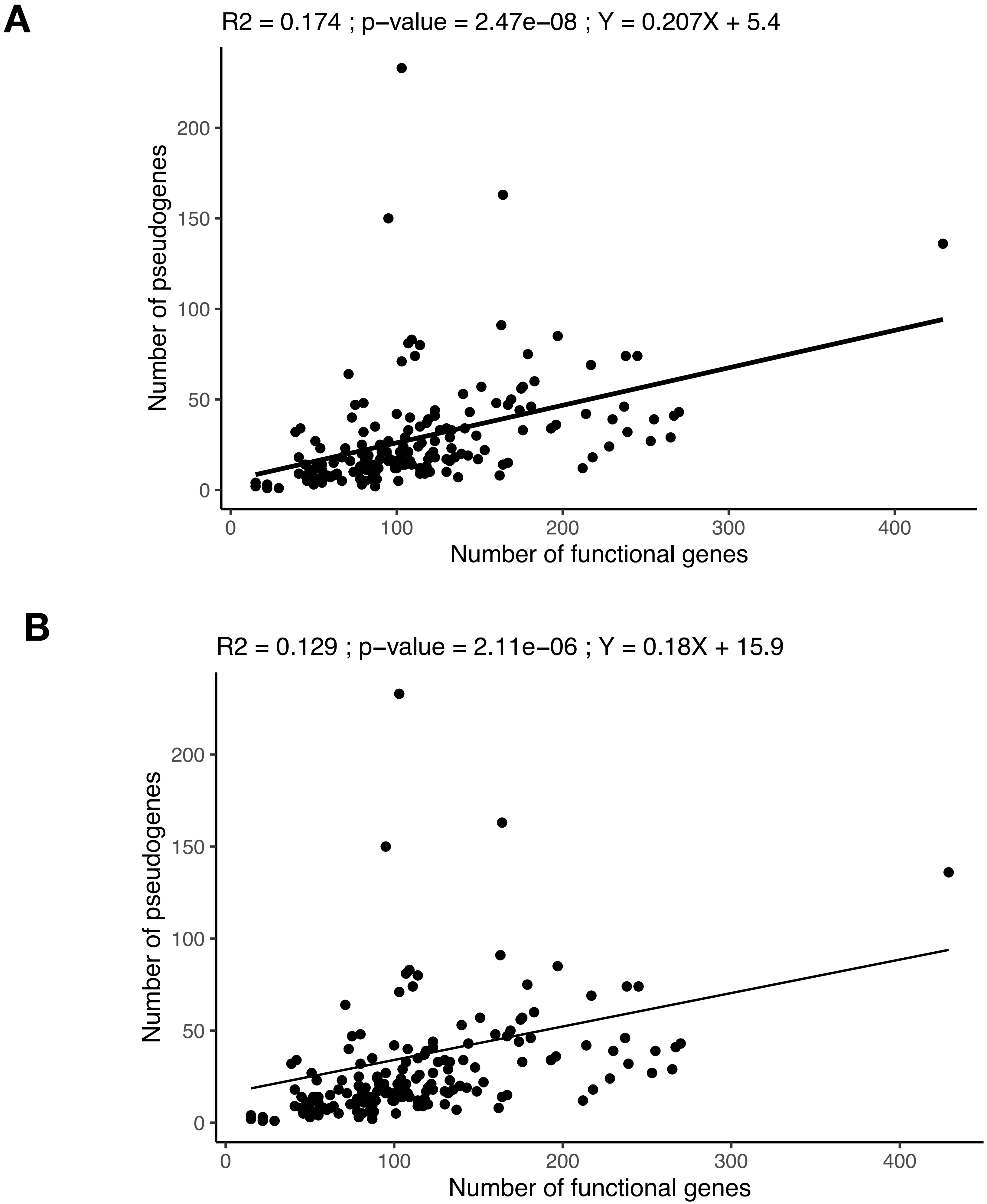
A
R2 = 0.174 ; p−value = 2.47e−08 ; Y = 0.207X + 5.4
Number of pseudogenes
Number of functional genes
0
50
100
150
200
0
100
200
300
400
B
R2 = 0.129 ; p−value = 2.11e−06 ; Y = 0.18X + 15.9
Number of pseudogenes
Number of functional genes
0
50
100
150
200
0
100
200
300
400

Fig. S3

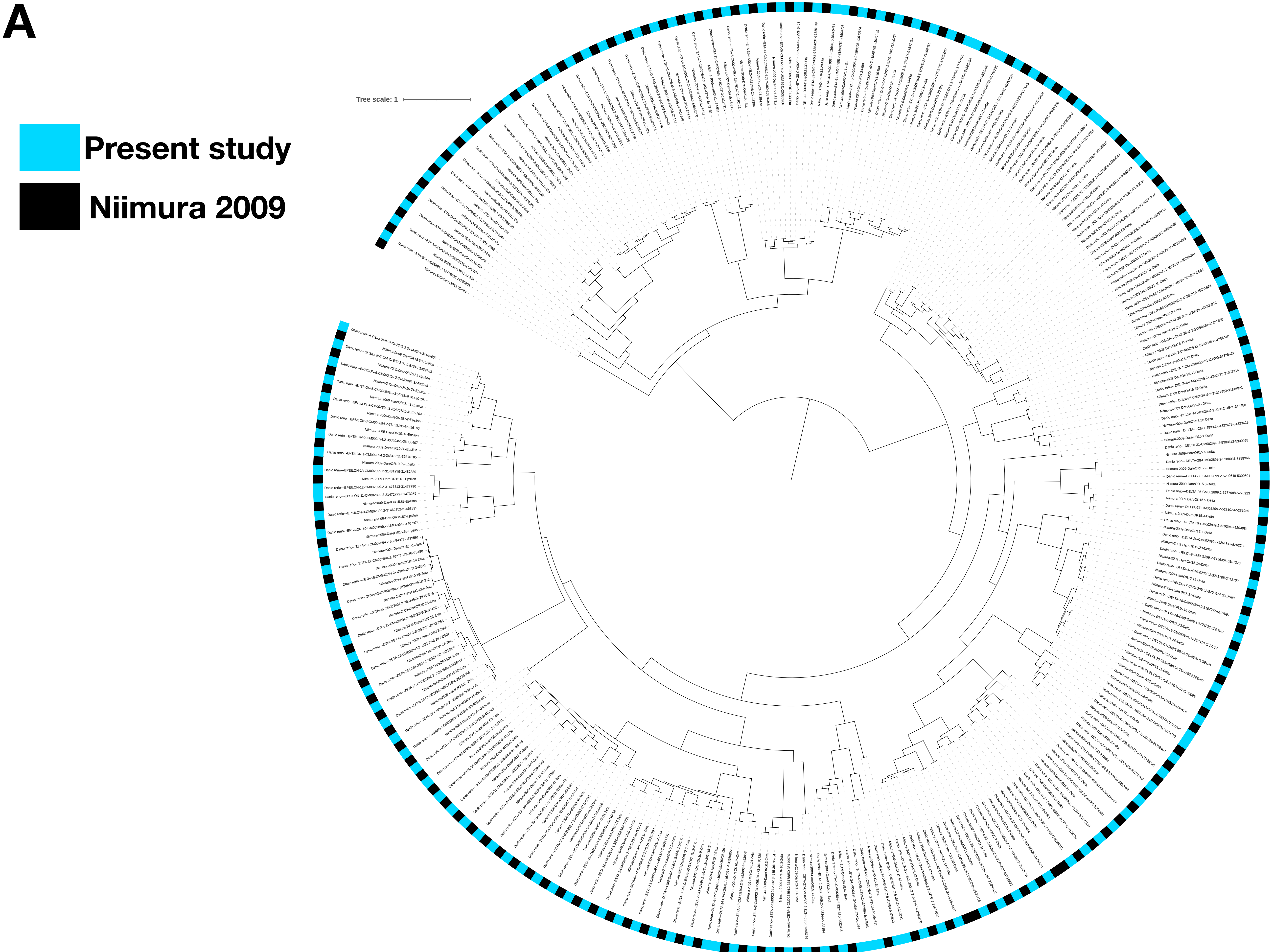

Fig. S4

B

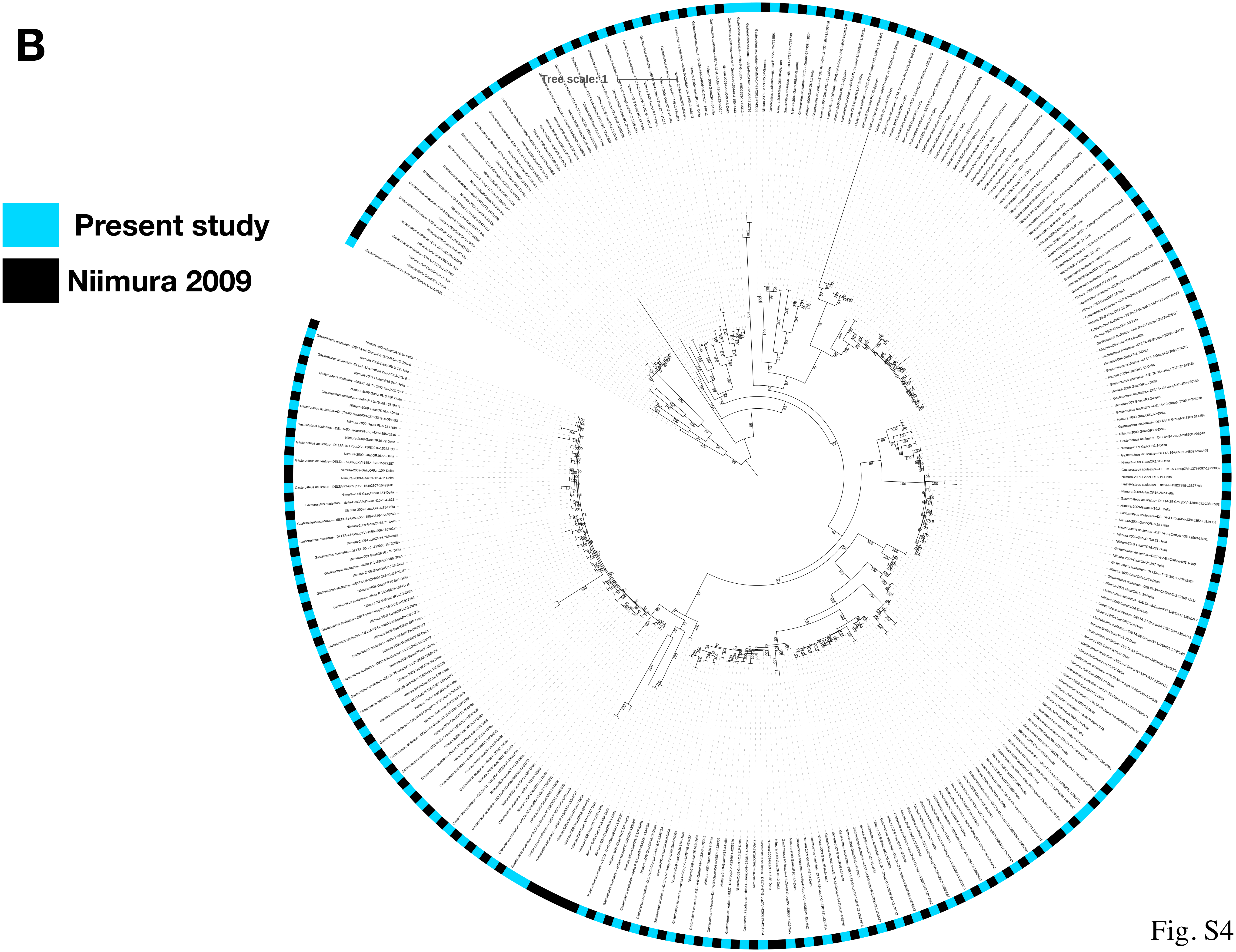

Fig. S4

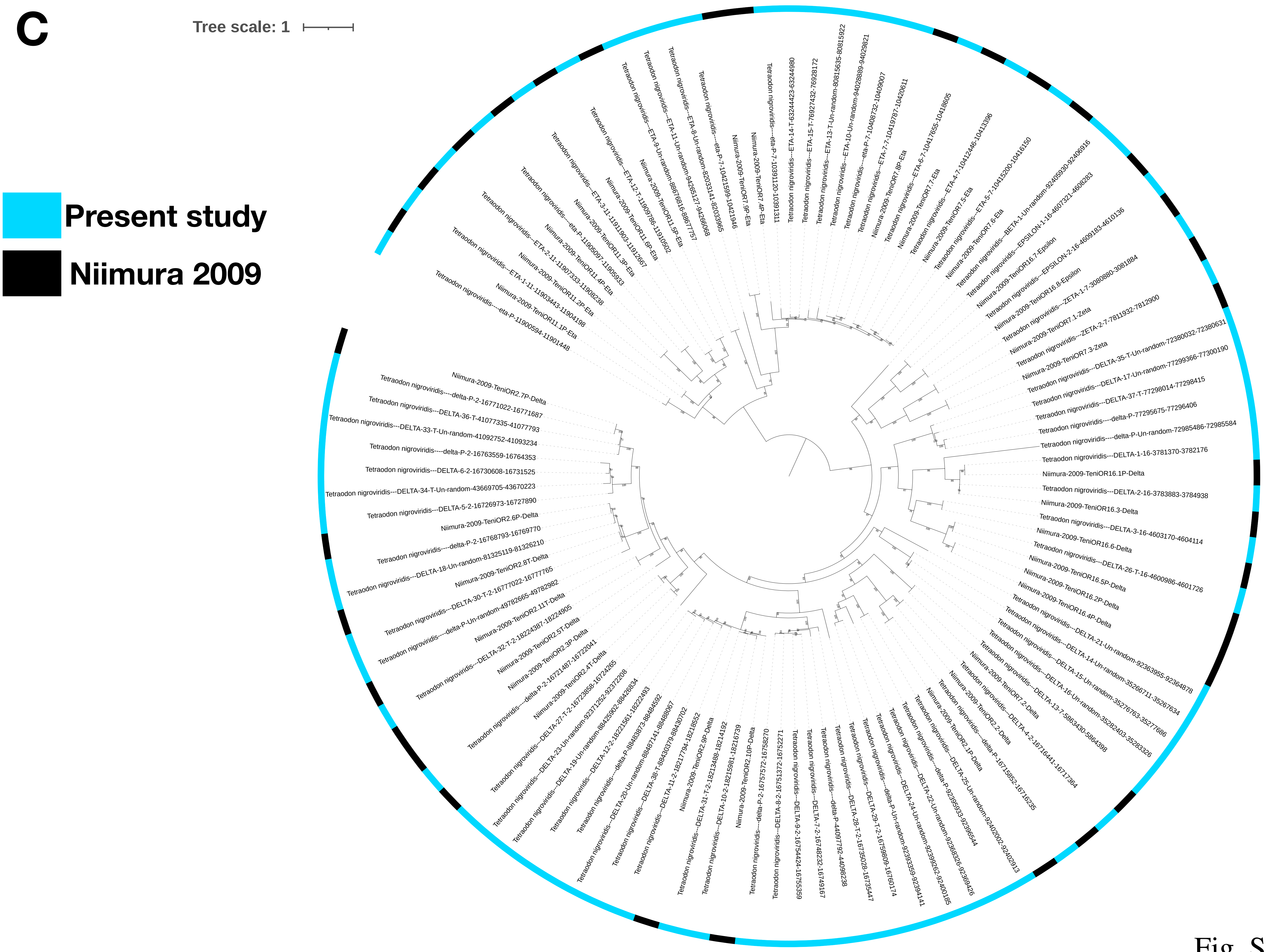
C
Tree scale: 1
Present study
Niimura 2009
Tetraodon nigroviridis----eta-P-11900594-11901448
Niimura-2009-TeniOR11.1P-Eta
Tetraodon nigroviridis---ETA-1-11-11903443-11904198
Niimura-2009-TeniOR11.2P-Eta
Tetraodon nigroviridis---ETA-2-11-11907333-11908238
Niimura-2009-TeniOR11.4P-Eta
Tetraodon nigroviridis----eta-P-11905097-11905933
Niimura-2009-TeniOR11.3P-Eta
Tetraodon nigroviridis---ETA-3-11-11911903-11912667
Niimura-2009-TeniOR11.6P-Eta
Tetraodon nigroviridis---ETA-12-T-11909786-11910502
Niimura-2009-TeniOR11.5P-Eta
Tetraodon nigroviridis---ETA-9-Un-random-88676816-88677757
Tetraodon nigroviridis---ETA-11-Un-random-94265127-94266068
Tetraodon nigroviridis---ETA-8-Un-random-82033141-82033965
Tetraodon nigroviridis----eta-P-7-10421599-10421946
Niimura-2009-TeniOR7.9P-Eta
Niimura-2009-TeniOR7.4P-Eta
Tetraodon nigroviridis----eta-P-7-10391120-10391311
Tetraodon nigroviridis---ETA-14-T-63244423-63244980
Tetraodon nigroviridis---ETA-15-T-76927432-76928172
Tetraodon nigroviridis---ETA-13-Un-random-80815635-80815922
Tetraodon nigroviridis---ETA-10-Un-random-94028889-94029821
Tetraodon nigroviridis----eta-P-7-10408732-10409007
Tetraodon nigroviridis---ETA-7-7-10419787-10420611
Niimura-2009-TeniOR7.8P-Eta
Tetraodon nigroviridis---ETA-6-7-10417655-10418605
Niimura-2009-TeniOR7.7-Eta
Tetraodon nigroviridis---ETA-4-7-10412446-10413396
Niimura-2009-TeniOR7.5-Eta
Tetraodon nigroviridis---ETA-5-7-10415200-10416150
Niimura-2009-TeniOR7.6-Eta
Tetraodon nigroviridis---BETA-1-Un-random-92405930-92406916
Niimura-2009-TeniOR16.7-Epsilon
Tetraodon nigroviridis---EPSILON-1-16-4607321-4608283
Niimura-2009-TeniOR16.8-Epsilon
Tetraodon nigroviridis---EPSILON-2-16-4609183-4610136
Tetraodon nigroviridis---ZETA-1-7-3080880-3081884
Niimura-2009-TeniOR7.1-Zeta
Tetraodon nigroviridis---ZETA-2-7-7811932-7812900
Niimura-2009-TeniOR7.3-Zeta
Tetraodon nigroviridis---DELTA-35-T-Un-random-72380032-72380631
Tetraodon nigroviridis---DELTA-17-Un-random-77299366-77300190
Tetraodon nigroviridis---DELTA-37-T-77298014-77298415
Tetraodon nigroviridis----delta-P-77295675-77296406
Tetraodon nigroviridis----delta-P-Un-random-72985486-72985584
Tetraodon nigroviridis---DELTA-1-16-3781370-3782176
Niimura-2009-TeniOR16.1P-Delta
Tetraodon nigroviridis---DELTA-2-16-3783883-3784938
Niimura-2009-TeniOR16.3-Delta
Tetraodon nigroviridis---DELTA-3-16-4603170-4604114
Niimura-2009-TeniOR16.6-Delta
Tetraodon nigroviridis---DELTA-26-T-16-4600986-4601726
Niimura-2009-TeniOR16.5P-Delta
Niimura-2009-TeniOR16.2P-Delta
Niimura-2009-TeniOR16.4P-Delta
Tetraodon nigroviridis---DELTA-21-Un-random-92363955-92364878
Tetraodon nigroviridis---DELTA-14-Un-random-35266711-35267634
Tetraodon nigroviridis---DELTA-15-Un-random-35276763-35277686
Tetraodon nigroviridis---DELTA-16-Un-random-35282403-35283326
Niimura-2009-TeniOR7.2-Delta
Tetraodon nigroviridis---DELTA-13-7-5863430-5864398
Niimura-2009-TeniOR2.2-Delta
Tetraodon nigroviridis---DELTA-4-2-16716441-16717364
Niimura-2009-TeniOR2.1P-Delta
Tetraodon nigroviridis----delta-P-16715852-16716235
Tetraodon nigroviridis---DELTA-25-Un-random-92402002-92402913
Tetraodon nigroviridis----delta-P-92395933-92396544
Tetraodon nigroviridis---DELTA-22-Un-random-92368326-92369426
Tetraodon nigroviridis---DELTA-24-Un-random-92399262-92400185
Tetraodon nigroviridis----delta-P-Un-random-92393359-92394141
Tetraodon nigroviridis---DELTA-29-T-2-16759809-16760174
Tetraodon nigroviridis---DELTA-28-T-2-16735028-16735447
Tetraodon nigroviridis----delta-P-44097792-44098238
Tetraodon nigroviridis---DELTA-7-2-16748232-16749167
Tetraodon nigroviridis---DELTA-9-2-16754424-16755359
Tetraodon nigroviridis---DELTA-8-2-16751372-16752271
Tetraodon nigroviridis----delta-P-2-16757572-16758270
Niimura-2009-TeniOR2.10P-Delta
Tetraodon nigroviridis---DELTA-10-2-18215981-18216739
Tetraodon nigroviridis---DELTA-31-T-2-18213488-18214192
Niimura-2009-TeniOR2.9P-Delta
Tetraodon nigroviridis---DELTA-11-2-18217794-18218652
Tetraodon nigroviridis---DELTA-38-T-88430379-88430702
Tetraodon nigroviridis---DELTA-20-Un-random-88487141-88488067
Tetraodon nigroviridis----delta-P-88483873-88484592
Tetraodon nigroviridis---DELTA-12-2-18221561-18222493
Tetraodon nigroviridis---DELTA-19-Un-random-88425902-88426834
Tetraodon nigroviridis---DELTA-23-Un-random-92371252-92372208
Tetraodon nigroviridis---DELTA-27-T-2-16723858-16724265
Niimura-2009-TeniOR2.4T-Delta
Tetraodon nigroviridis----delta-P-2-16721487-16722041
Niimura-2009-TeniOR2.3P-Delta
Niimura-2009-TeniOR2.5T-Delta
Tetraodon nigroviridis---DELTA-32-T-2-18224387-18224905
Niimura-2009-TeniOR2.11T-Delta
Tetraodon nigroviridis----delta-P-Un-random-49782665-49782982
Tetraodon nigroviridis---DELTA-30-T-2-16777022-16777765
Niimura-2009-TeniOR2.8T-Delta
Tetraodon nigroviridis---DELTA-18-Un-random-81325119-81326210
Tetraodon nigroviridis----delta-P-2-16768793-16769770
Niimura-2009-TeniOR2.6P-Delta
Tetraodon nigroviridis---DELTA-5-2-16726973-16727890
Tetraodon nigroviridis---DELTA-34-T-Un-random-43669705-43670223
Tetraodon nigroviridis---DELTA-6-2-16730608-16731525
Tetraodon nigroviridis----delta-P-2-16763559-16764353
Tetraodon nigroviridis---DELTA-33-T-Un-random-41092752-41093234
Tetraodon nigroviridis---DELTA-36-T-41077335-41077793
Tetraodon nigroviridis----delta-P-2-16771022-16771687
Niimura-2009-TeniOR2.7P-Delta

Fig. S4

D

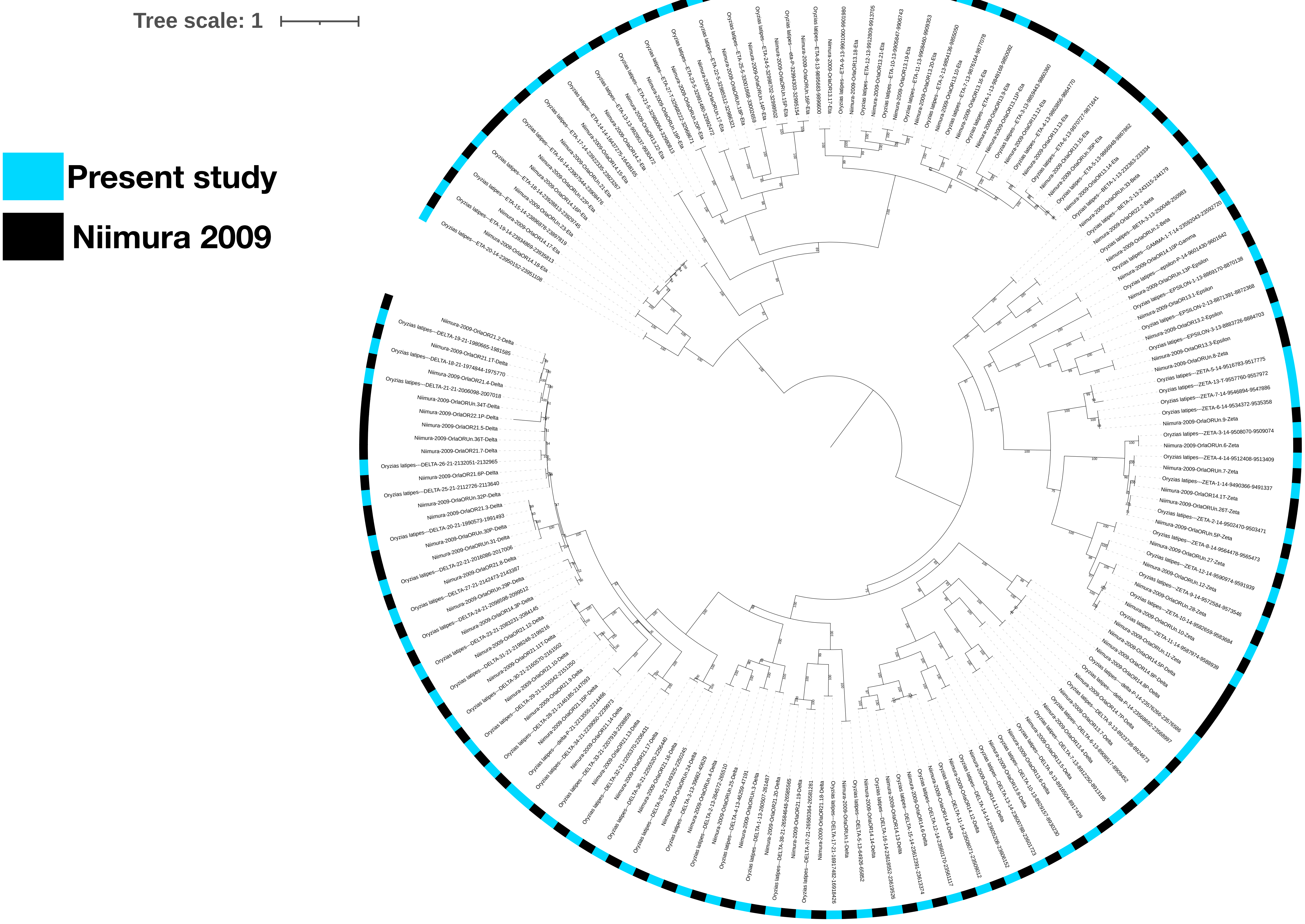

Fig. S4

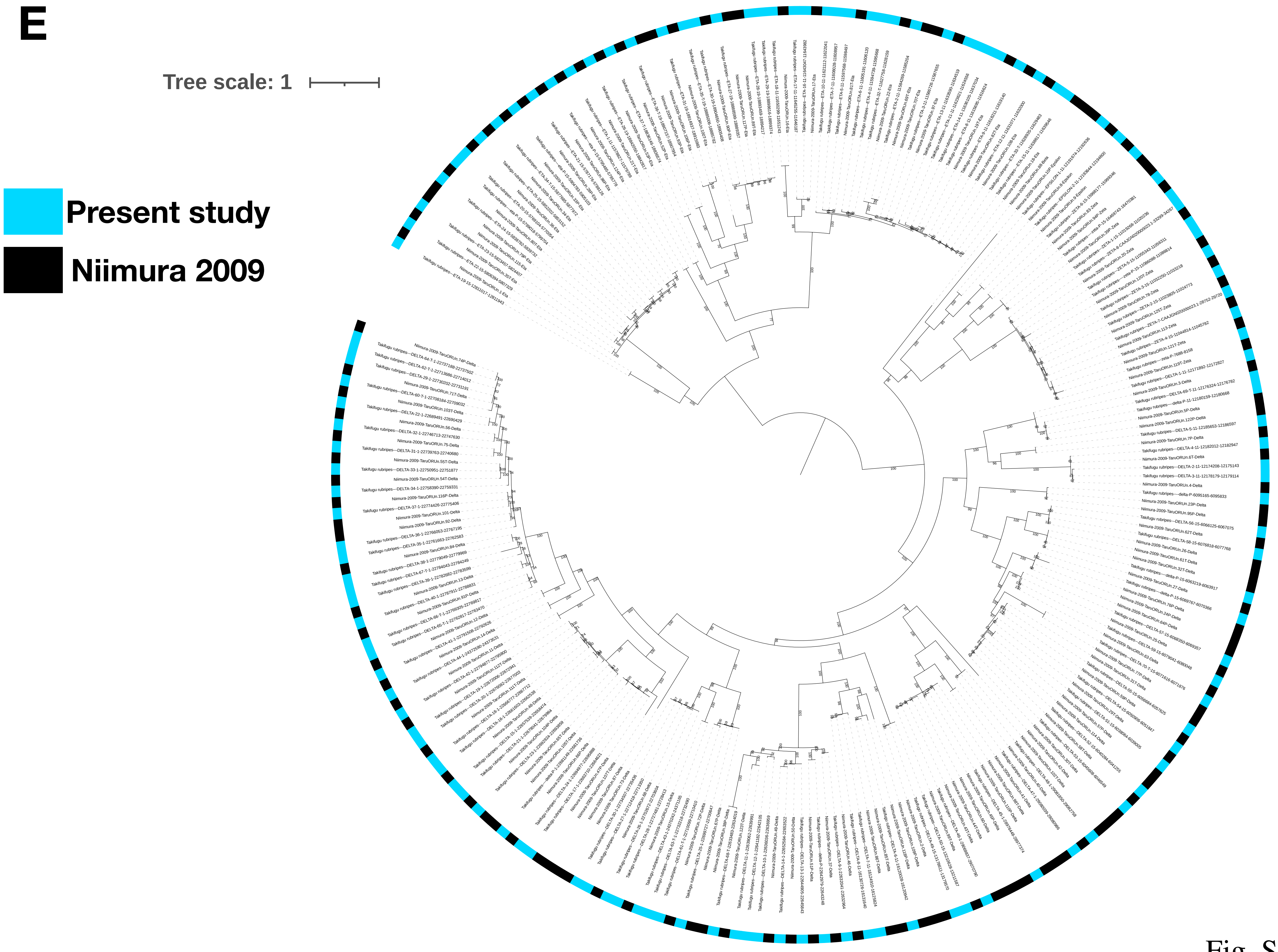

Fig. S4

F

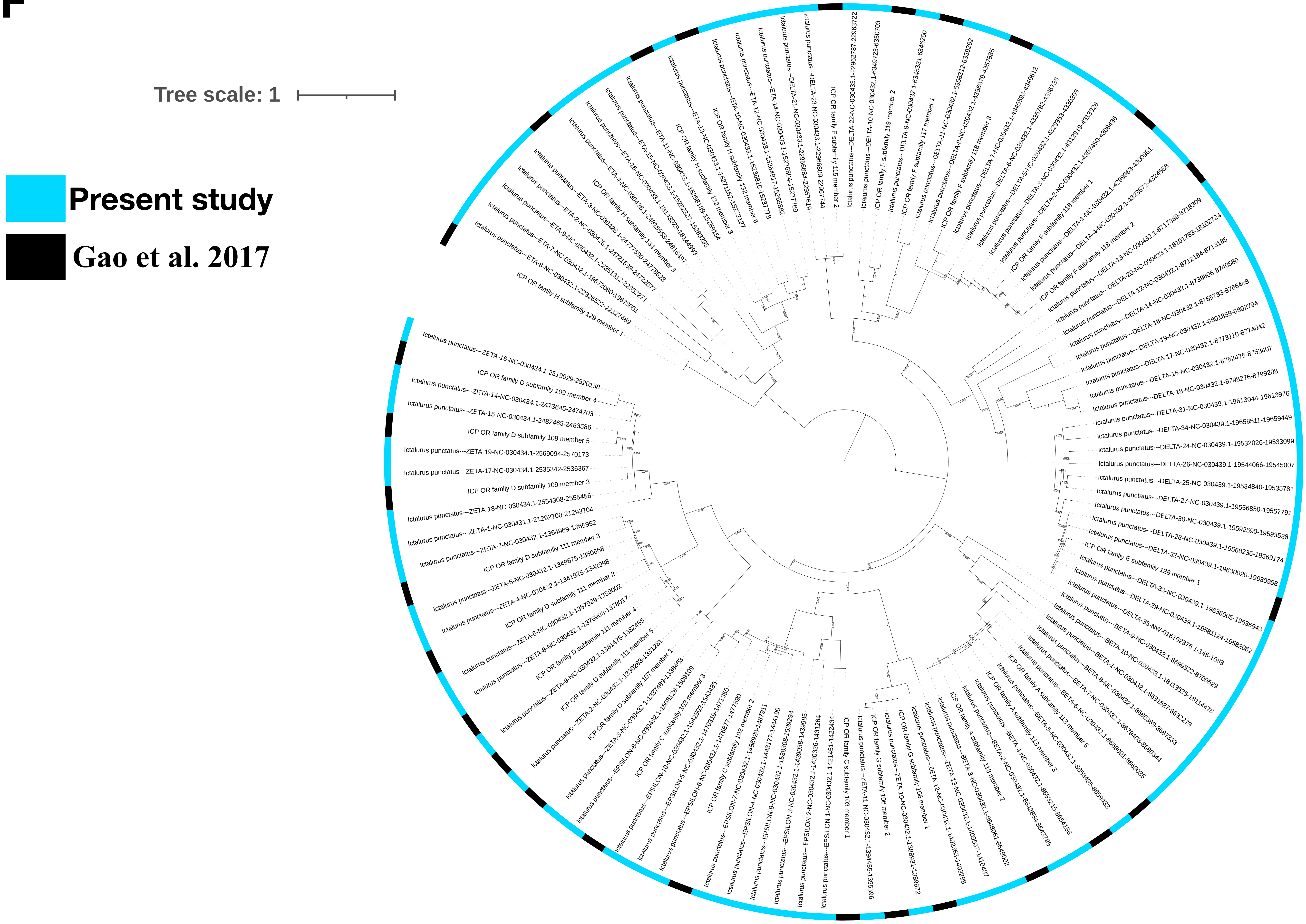
Tree scale: 1
Present study
Gao et al. 2017
ICP OR family H subfamily 129 member 1
Ictalurus punctatus---ETA-8-NC-030432.1-22326522-22327469
Ictalurus punctatus---ETA-7-NC-030432.1-19672080-19673051
Ictalurus punctatus---ETA-9-NC-030432.1-22351312-22352271
Ictalurus punctatus---ETA-2-NC-030426.1-24721639-24722577
Ictalurus punctatus---ETA-3-NC-030426.1-24777590-24778528
Ictalurus punctatus---ETA-4-NC-030426.1-24815553-24816497
ICP OR family H subfamily 134 member 3
Ictalurus punctatus---ETA-16-NC-030433.1-18143929-18144993
Ictalurus punctatus---ETA-15-NC-030433.1-15282327-15283295
Ictalurus punctatus---ETA-11-NC-030433.1-15258189-15259154
ICP OR family H subfamily 132 member 3
Ictalurus punctatus---ETA-13-NC-030433.1-15271162-15272127
ICP OR family H subfamily 132 member 6
Ictalurus punctatus---ETA-10-NC-030433.1-15236816-15237778
Ictalurus punctatus---ETA-12-NC-030433.1-15264917-15265882
Ictalurus punctatus---ETA-14-NC-030433.1-15276804-15277769
Ictalurus punctatus---DELTA-21-NC-030433.1-22956684-22957619
Ictalurus punctatus---DELTA-23-NC-030433.1-22966809-22967744
ICP OR family F subfamily 115 member 2
Ictalurus punctatus---DELTA-22-NC-030433.1-22962787-22963722
Ictalurus punctatus---DELTA-10-NC-030432.1-6349723-6350703
ICP OR family F subfamily 119 member 2
Ictalurus punctatus---DELTA-9-NC-030432.1-6345331-6346260
ICP OR family F subfamily 117 member 1
Ictalurus punctatus---DELTA-11-NC-030432.1-6358312-6359262
ICP OR family F subfamily 118 member 3
Ictalurus punctatus---DELTA-8-NC-030432.1-4356879-4357835
Ictalurus punctatus---DELTA-7-NC-030432.1-4345593-4346612
Ictalurus punctatus---DELTA-6-NC-030432.1-4335782-4336738
Ictalurus punctatus---DELTA-5-NC-030432.1-4329353-4330309
Ictalurus punctatus---DELTA-3-NC-030432.1-4312919-4313926
Ictalurus punctatus---DELTA-2-NC-030432.1-4307450-4308436
ICP OR family F subfamily 118 member 1
Ictalurus punctatus---DELTA-1-NC-030432.1-4299963-4300961
Ictalurus punctatus---DELTA-4-NC-030432.1-4323572-4324558
ICP OR family F subfamily 118 member 2
Ictalurus punctatus---DELTA-13-NC-030432.1-8717389-8718309
Ictalurus punctatus---DELTA-20-NC-030433.1-18101783-18102724
Ictalurus punctatus---DELTA-12-NC-030432.1-8712184-8713185
Ictalurus punctatus---DELTA-14-NC-030432.1-8739606-8740580
Ictalurus punctatus---DELTA-16-NC-030432.1-8765733-8766488
Ictalurus punctatus---DELTA-19-NC-030432.1-8801859-8802794
Ictalurus punctatus---DELTA-17-NC-030432.1-8773110-8774042
Ictalurus punctatus---DELTA-15-NC-030432.1-8752475-8753407
Ictalurus punctatus---DELTA-18-NC-030432.1-8798276-8799208
Ictalurus punctatus---DELTA-31-NC-030439.1-19613044-19613976
Ictalurus punctatus---DELTA-34-NC-030439.1-19658511-19659449
Ictalurus punctatus---DELTA-24-NC-030439.1-19532026-19533099
Ictalurus punctatus---DELTA-26-NC-030439.1-19544066-19545007
Ictalurus punctatus---DELTA-25-NC-030439.1-19534840-19535781
Ictalurus punctatus---DELTA-27-NC-030439.1-19556850-19557791
Ictalurus punctatus---DELTA-30-NC-030439.1-19592590-19593528
Ictalurus punctatus---DELTA-28-NC-030439.1-19568236-19569174
ICP OR family E subfamily 128 member 1
Ictalurus punctatus---DELTA-32-NC-030439.1-19630020-19630958
Ictalurus punctatus---DELTA-33-NC-030439.1-19636005-19636943
Ictalurus punctatus---DELTA-29-NC-030439.1-19581124-19582062
Ictalurus punctatus---DELTA-35-NW-016102376.1-145-1083
Ictalurus punctatus---BETA-9-NC-030432.1-8699522-8700529
Ictalurus punctatus---BETA-10-NC-030433.1-18113525-18114478
Ictalurus punctatus---BETA-1-NC-030432.1-8631527-8632279
Ictalurus punctatus---BETA-8-NC-030432.1-8686389-8687333
Ictalurus punctatus---BETA-7-NC-030432.1-8679403-8680344
Ictalurus punctatus---BETA-6-NC-030432.1-8668091-8669035
ICP OR family A subfamily 113 member 5
Ictalurus punctatus---BETA-5-NC-030432.1-8658495-8659433
ICP OR family A subfamily 113 member 3
Ictalurus punctatus---BETA-4-NC-030432.1-8653215-8654156
Ictalurus punctatus---BETA-2-NC-030432.1-8642854-8643795
ICP OR family A subfamily 113 member 2
Ictalurus punctatus---BETA-3-NC-030432.1-8648061-8649002
Ictalurus punctatus---ZETA-13-NC-030432.1-1409537-1410487
Ictalurus punctatus---ZETA-12-NC-030432.1-1402363-1403298
ICP OR family G subfamily 106 member 1
Ictalurus punctatus---ZETA-10-NC-030432.1-1388931-1389872
ICP OR family G subfamily 106 member 2
Ictalurus punctatus---ZETA-11-NC-030432.1-1394455-1395396
ICP OR family C subfamily 103 member 1
Ictalurus punctatus---EPSILON-1-NC-030432.1-1421451-1422434
Ictalurus punctatus---EPSILON-2-NC-030432.1-1430326-1431264
Ictalurus punctatus---EPSILON-3-NC-030432.1-1439038-1439985
Ictalurus punctatus---EPSILON-9-NC-030432.1-1538308-1539294
Ictalurus punctatus---EPSILON-4-NC-030432.1-1443177-1444190
Ictalurus punctatus---EPSILON-7-NC-030432.1-1486928-1487911
ICP OR family C subfamily 102 member 2
Ictalurus punctatus---EPSILON-6-NC-030432.1-1476877-1477890
Ictalurus punctatus---EPSILON-5-NC-030432.1-1470319-1471350
Ictalurus punctatus---EPSILON-10-NC-030432.1-1542502-1543485
ICP OR family C subfamily 102 member 3
Ictalurus punctatus---EPSILON-8-NC-030432.1-1508126-1509109
Ictalurus punctatus---ZETA-3-NC-030432.1-1337489-1338463
ICP OR family D subfamily 107 member 1
Ictalurus punctatus---ZETA-2-NC-030432.1-1330283-1331281
ICP OR family D subfamily 111 member 5
Ictalurus punctatus---ZETA-9-NC-030432.1-1381475-1382455
ICP OR family D subfamily 111 member 4
Ictalurus punctatus---ZETA-8-NC-030432.1-1376908-1378017
Ictalurus punctatus---ZETA-6-NC-030432.1-1357929-1359002
ICP OR family D subfamily 111 member 2
Ictalurus punctatus---ZETA-4-NC-030432.1-1341925-1342998
Ictalurus punctatus---ZETA-5-NC-030432.1-1349675-1350658
ICP OR family D subfamily 111 member 3
Ictalurus punctatus---ZETA-7-NC-030432.1-1364969-1365952
Ictalurus punctatus---ZETA-1-NC-030431.1-21292700-21293704
Ictalurus punctatus---ZETA-18-NC-030434.1-2554308-2555456
ICP OR family D subfamily 109 member 3
Ictalurus punctatus---ZETA-17-NC-030434.1-2535342-2536367
Ictalurus punctatus---ZETA-19-NC-030434.1-2569094-2570173
ICP OR family D subfamily 109 member 5
Ictalurus punctatus---ZETA-15-NC-030434.1-2482465-2483586
Ictalurus punctatus---ZETA-14-NC-030434.1-2473645-2474703
ICP OR family D subfamily 109 member 4
Ictalurus punctatus---ZETA-16-NC-030434.1-2519029-2520138

Fig. S4

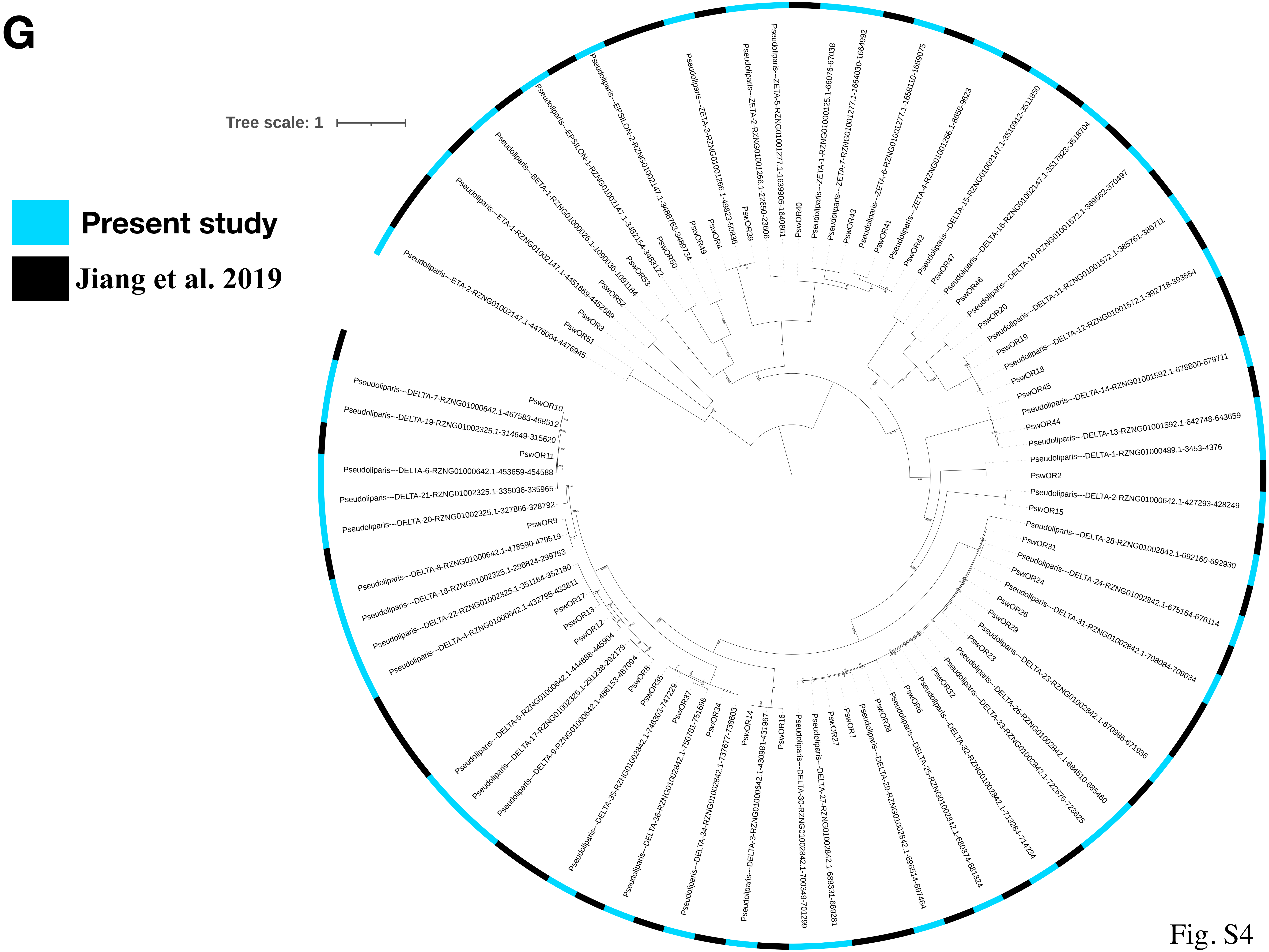
G
Tree scale: 1
Present study
Jiang et al. 2019
Pseudoliparis---ETA-2-RZNG01002147.1-4476004-4476945
PswOR51
Pseudoliparis---ETA-1-RZNG01002147.1-4451669-4452589
PswOR3
Pseudoliparis---BETA-1-RZNG01000026.1-1090036-1091184
PswOR52
Pseudoliparis---EPSILON-1-RZNG01002147.1-3482154-3483122
PswOR53
Pseudoliparis---EPSILON-2-RZNG01002147.1-3488763-3489734
PswOR50
PswOR49
Pseudoliparis---ZETA-3-RZNG01001266.1-49823-50836
PswOR4
Pseudoliparis---ZETA-2-RZNG01001266.1-22650-23606
PswOR39
Pseudoliparis---ZETA-5-RZNG01001277.1-1639905-1640861
PswOR40
Pseudoliparis---ZETA-1-RZNG01000125.1-66076-67038
Pseudoliparis---ZETA-7-RZNG01001277.1-1664030-1664992
PswOR43
Pseudoliparis---ZETA-6-RZNG01001277.1-1658110-1659075
PswOR41
Pseudoliparis---ZETA-4-RZNG01001266.1-8658-9623
PswOR42
Pseudoliparis---DELTA-15-RZNG01002147.1-3510912-3511850
PswOR47
Pseudoliparis---DELTA-16-RZNG01002147.1-3517823-3518704
PswOR46
Pseudoliparis---DELTA-10-RZNG01001572.1-369562-370497
PswOR20
Pseudoliparis---DELTA-11-RZNG01001572.1-385761-386711
PswOR19
Pseudoliparis---DELTA-12-RZNG01001572.1-392718-393554
PswOR18
PswOR45
Pseudoliparis---DELTA-14-RZNG01001592.1-678800-679711
PswOR44
Pseudoliparis---DELTA-13-RZNG01001592.1-642748-643659
Pseudoliparis---DELTA-1-RZNG01000489.1-3453-4376
PswOR2
Pseudoliparis---DELTA-2-RZNG01000642.1-427293-428249
PswOR15
Pseudoliparis---DELTA-28-RZNG01002842.1-692160-692930
PswOR31
Pseudoliparis---DELTA-24-RZNG01002842.1-675164-676114
PswOR24
Pseudoliparis---DELTA-31-RZNG01002842.1-708084-709034
PswOR26
PswOR29
Pseudoliparis---DELTA-23-RZNG01002842.1-670986-671936
PswOR23
Pseudoliparis---DELTA-26-RZNG01002842.1-684510-685460
Pseudoliparis---DELTA-33-RZNG01002842.1-722675-723625
PswOR32
Pseudoliparis---DELTA-32-RZNG01002842.1-713284-714234
PswOR6
Pseudoliparis---DELTA-25-RZNG01002842.1-680374-681324
PswOR28
Pseudoliparis---DELTA-29-RZNG01002842.1-696514-697464
PswOR7
PswOR27
Pseudoliparis---DELTA-27-RZNG01002842.1-688331-689281
Pseudoliparis---DELTA-30-RZNG01002842.1-700349-701299
PswOR16
Pseudoliparis---DELTA-3-RZNG01000642.1-430981-431967
PswOR14
Pseudoliparis---DELTA-34-RZNG01002842.1-737677-738603
PswOR34
Pseudoliparis---DELTA-36-RZNG01002842.1-750781-751698
PswOR37
Pseudoliparis---DELTA-35-RZNG01002842.1-746303-747229
PswOR35
PswOR8
Pseudoliparis---DELTA-9-RZNG01000642.1-486153-487094
Pseudoliparis---DELTA-17-RZNG01002325.1-291238-292179
Pseudoliparis---DELTA-5-RZNG01000642.1-444888-445904
PswOR12
PswOR13
PswOR17
Pseudoliparis---DELTA-4-RZNG01000642.1-432795-433811
Pseudoliparis---DELTA-22-RZNG01002325.1-351164-352180
Pseudoliparis---DELTA-18-RZNG01002325.1-298824-299753
Pseudoliparis---DELTA-8-RZNG01000642.1-478590-479519
PswOR9
Pseudoliparis---DELTA-20-RZNG01002325.1-327866-328792
Pseudoliparis---DELTA-21-RZNG01002325.1-335036-335965
Pseudoliparis---DELTA-6-RZNG01000642.1-453659-454588
PswOR11
Pseudoliparis---DELTA-19-RZNG01002325.1-314649-315620
PswOR10
Pseudoliparis---DELTA-7-RZNG01000642.1-467583-468512

Fig. S4

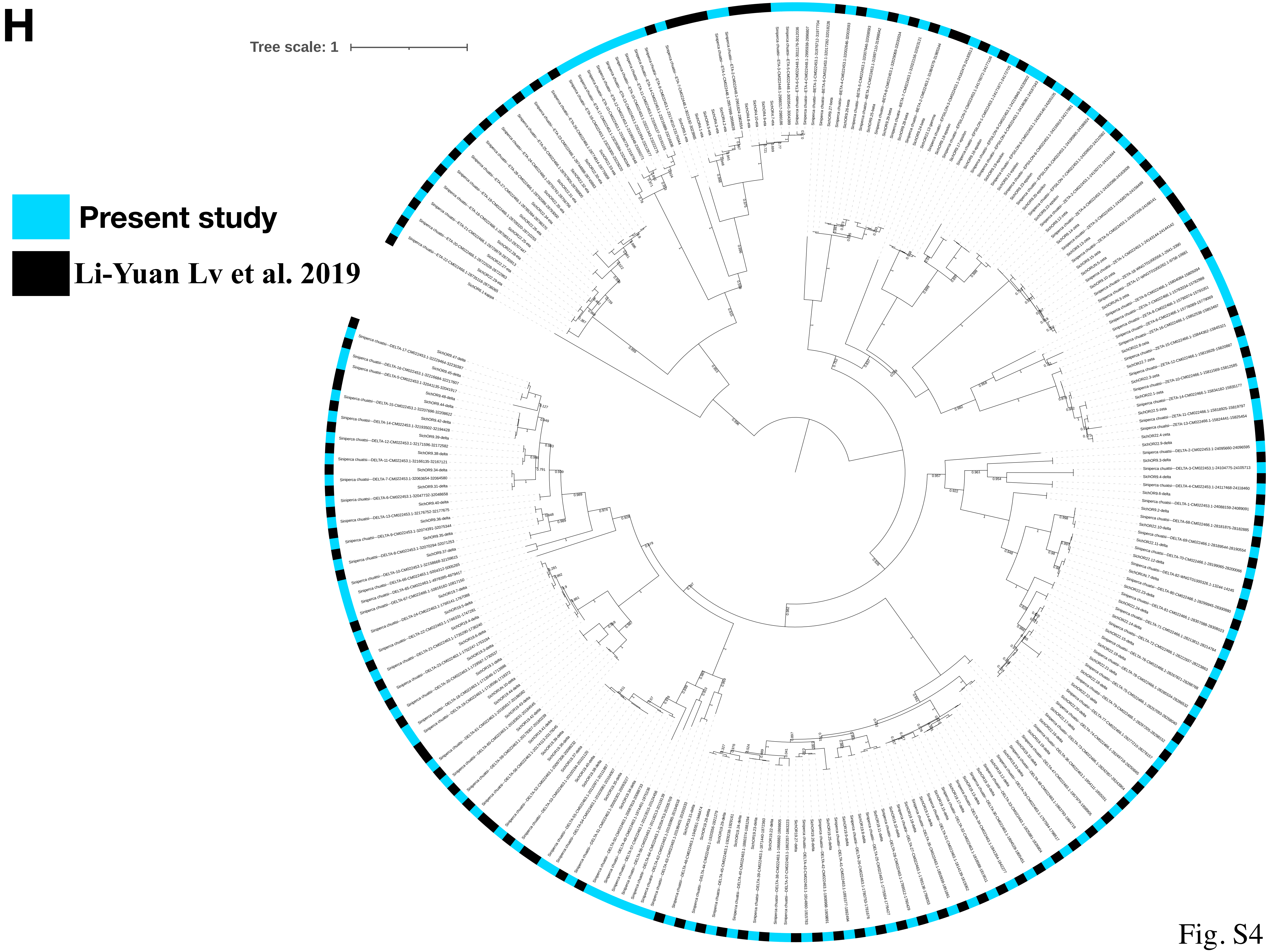

Fig. S4

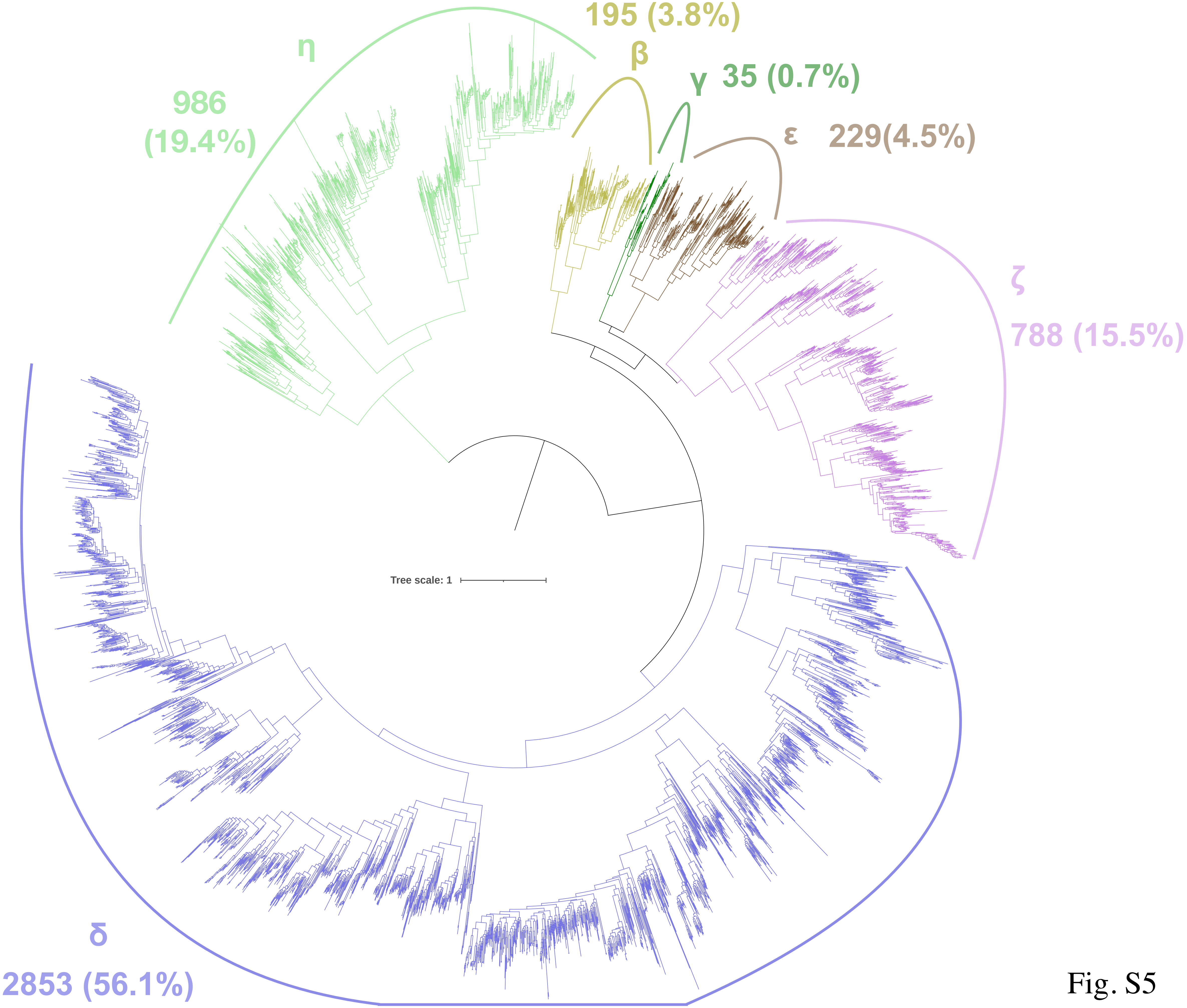

Fig. S5

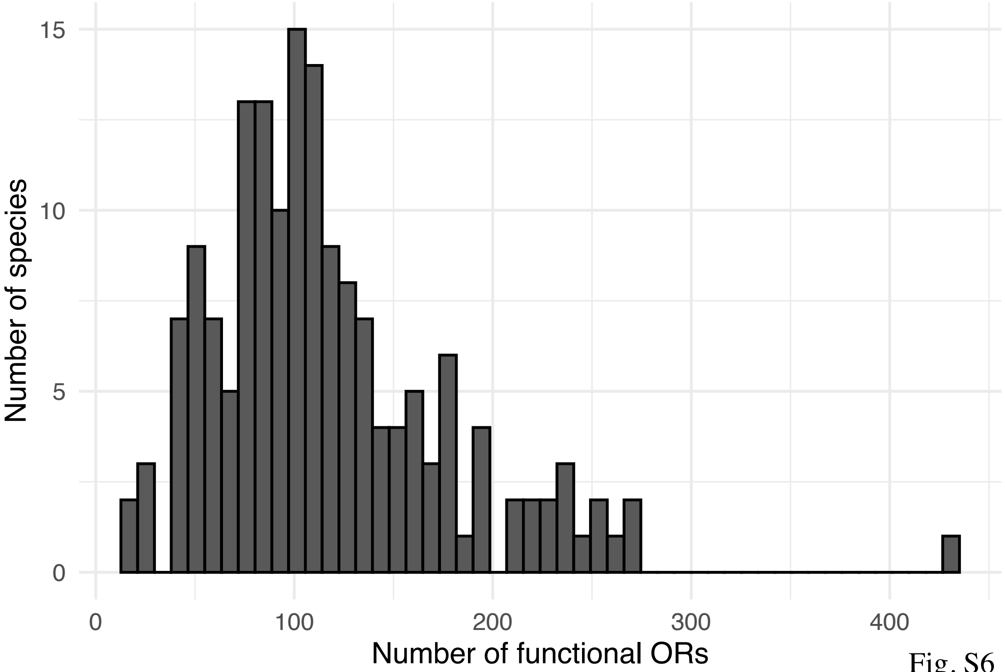

Number of species
15
10
5
0
0
100
200
300
400
Number of functional ORs

Fig. S6

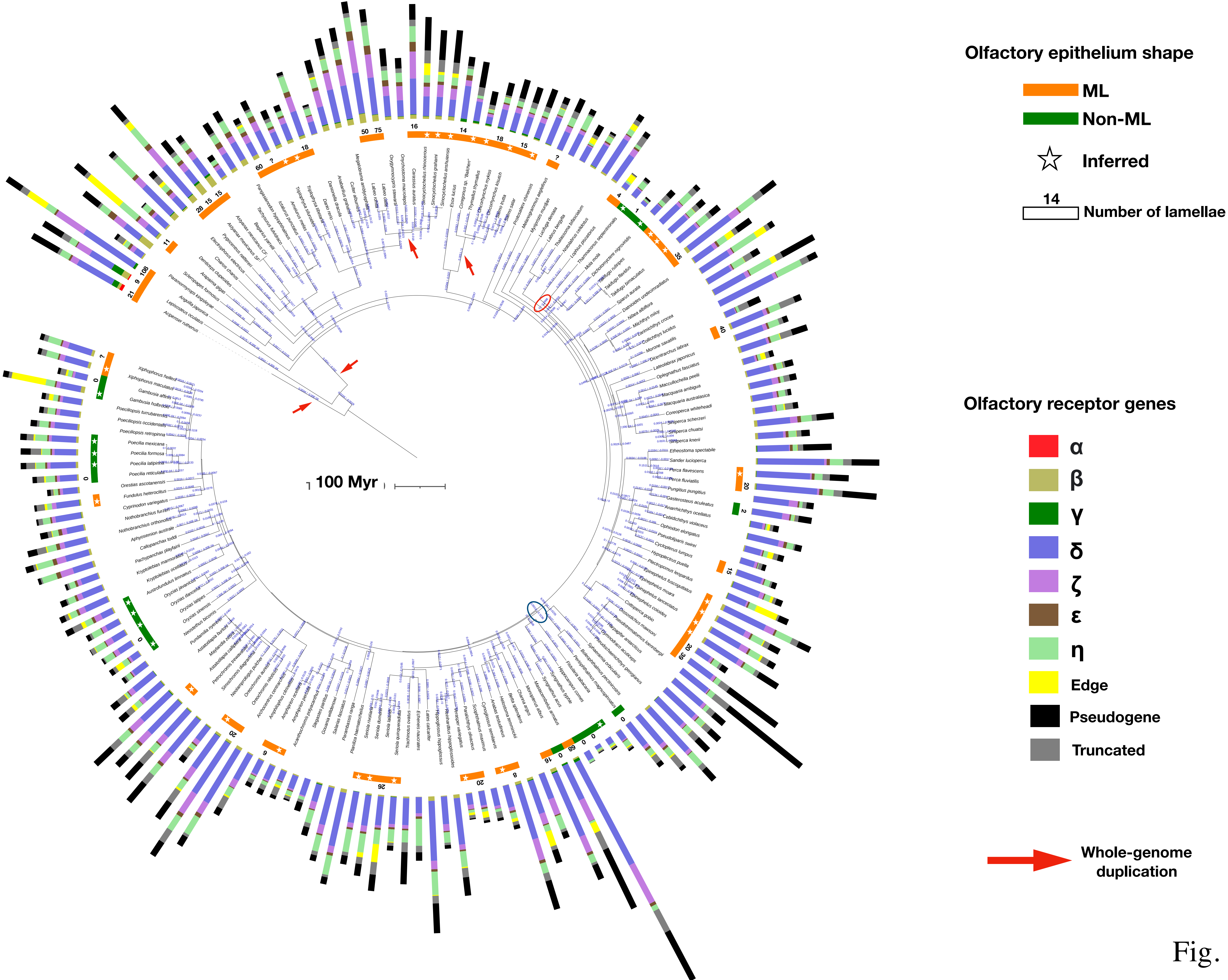
Olfactory epithelium shape
ML
Non-ML
Inferred
14
Number of lamellae
Olfactory receptor genes
α
β
γ
δ
ζ
ε
η
Edge
Pseudogene
Truncated
Whole-genome duplication
100 Myr

Fig. S7

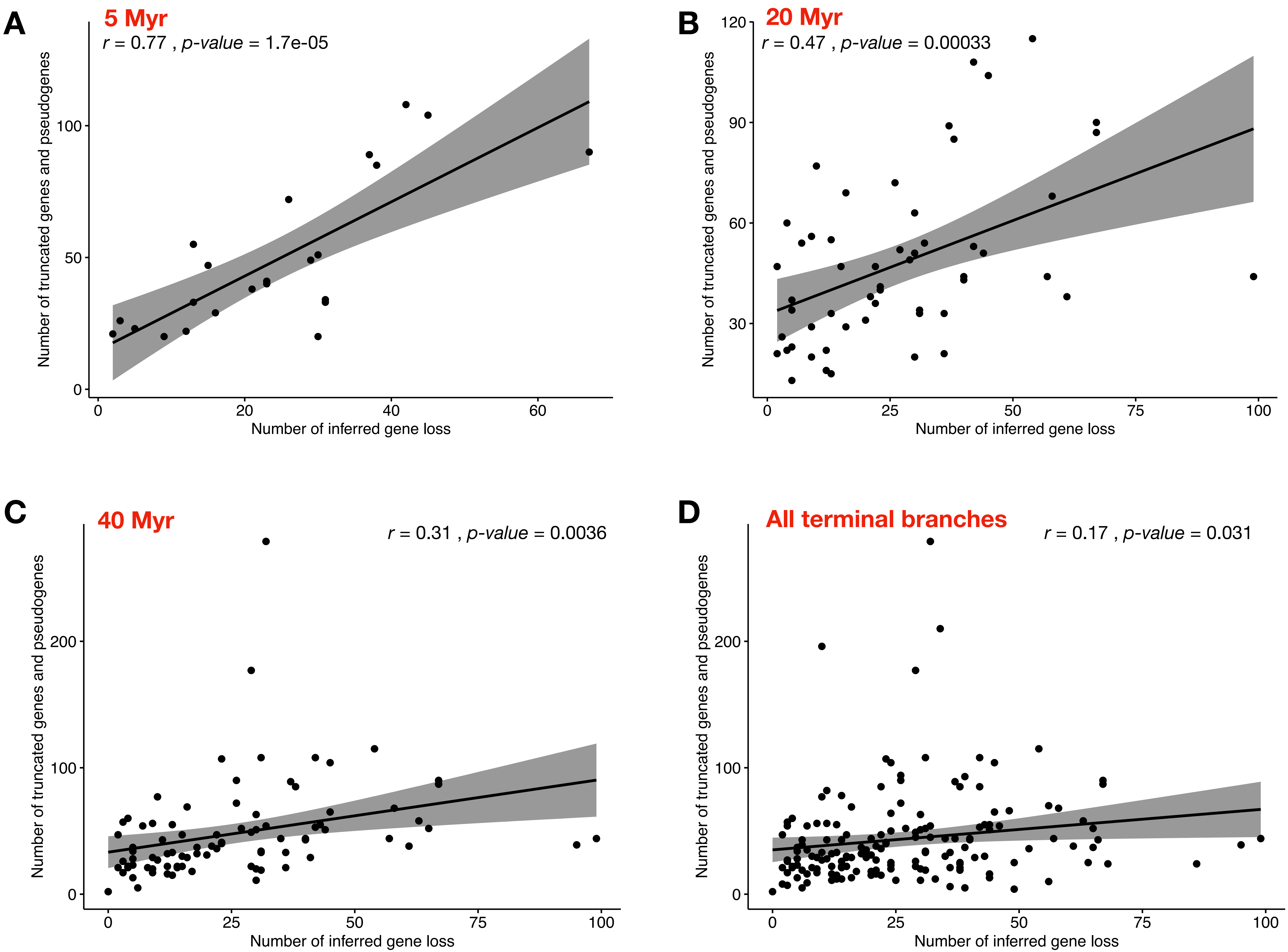

A
5 Myr
r = 0.77 , p-value = 1.7e-05
Number of truncated genes and pseudogenes
Number of inferred gene loss
B
20 Myr
r = 0.47 , p-value = 0.00033
Number of truncated genes and pseudogenes
Number of inferred gene loss
C
40 Myr
r = 0.31 , p-value = 0.0036
Number of truncated genes and pseudogenes
Number of inferred gene loss
D
All terminal branches
r = 0.17 , p-value = 0.031
Number of truncated genes and pseudogenes
Number of inferred gene loss

Fig. S8

A- Pseudogenes

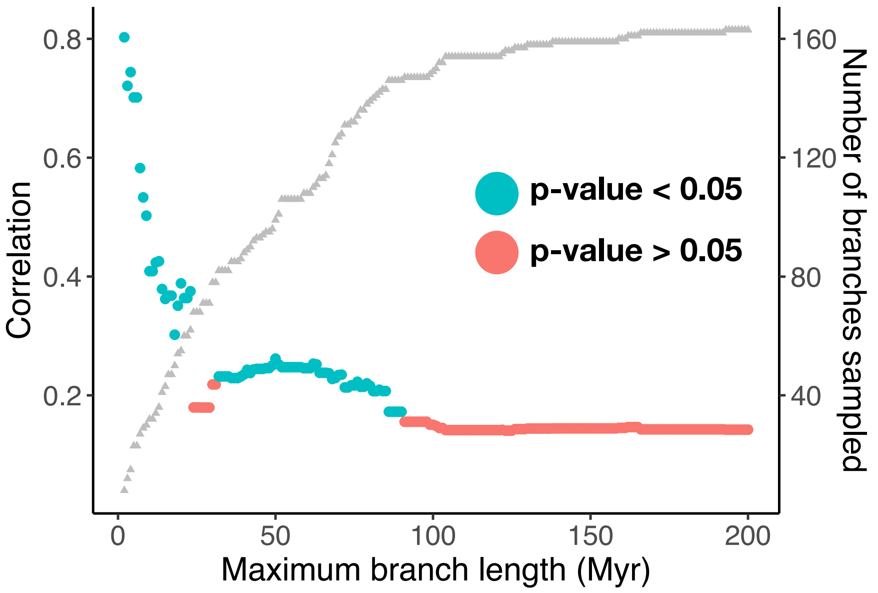

B- Truncated genes

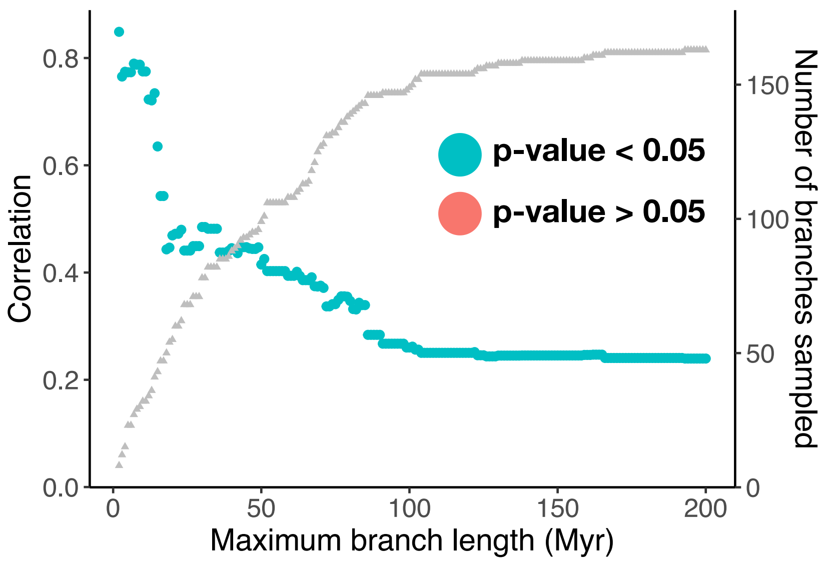

C- Edge genes

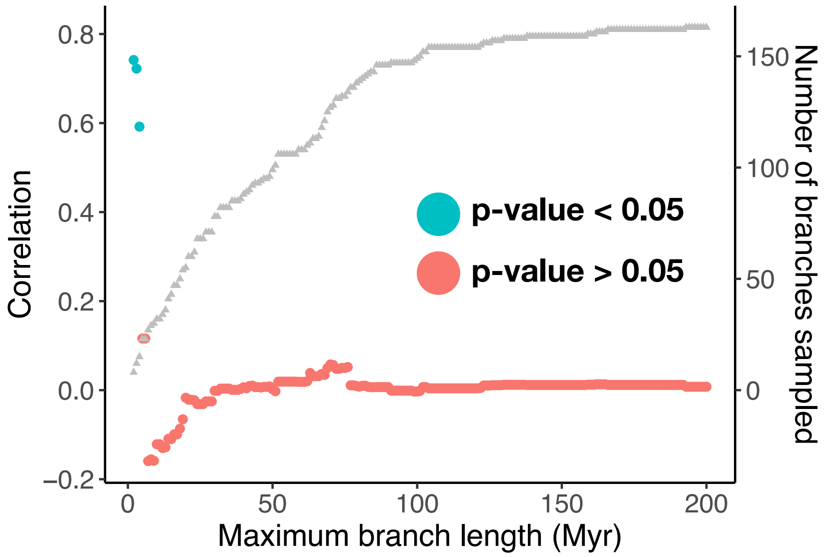

Fig. S9

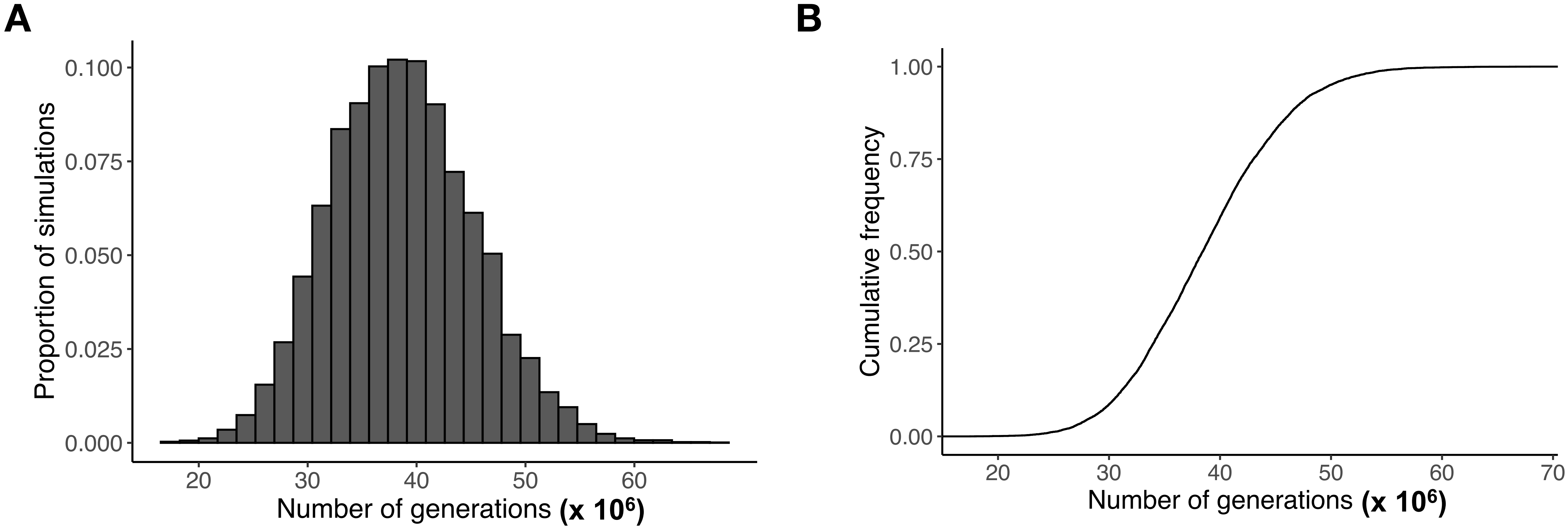
A
Proportion of simulations
0.100
0.075
0.050
0.025
0.000
20
30
40
50
60
Number of generations (x 10^6)
B
Cumulative frequency
1.00
0.75
0.50
0.25
0.00
20
30
40
50
60
70
Number of generations (x 10^6)

Fig. S10

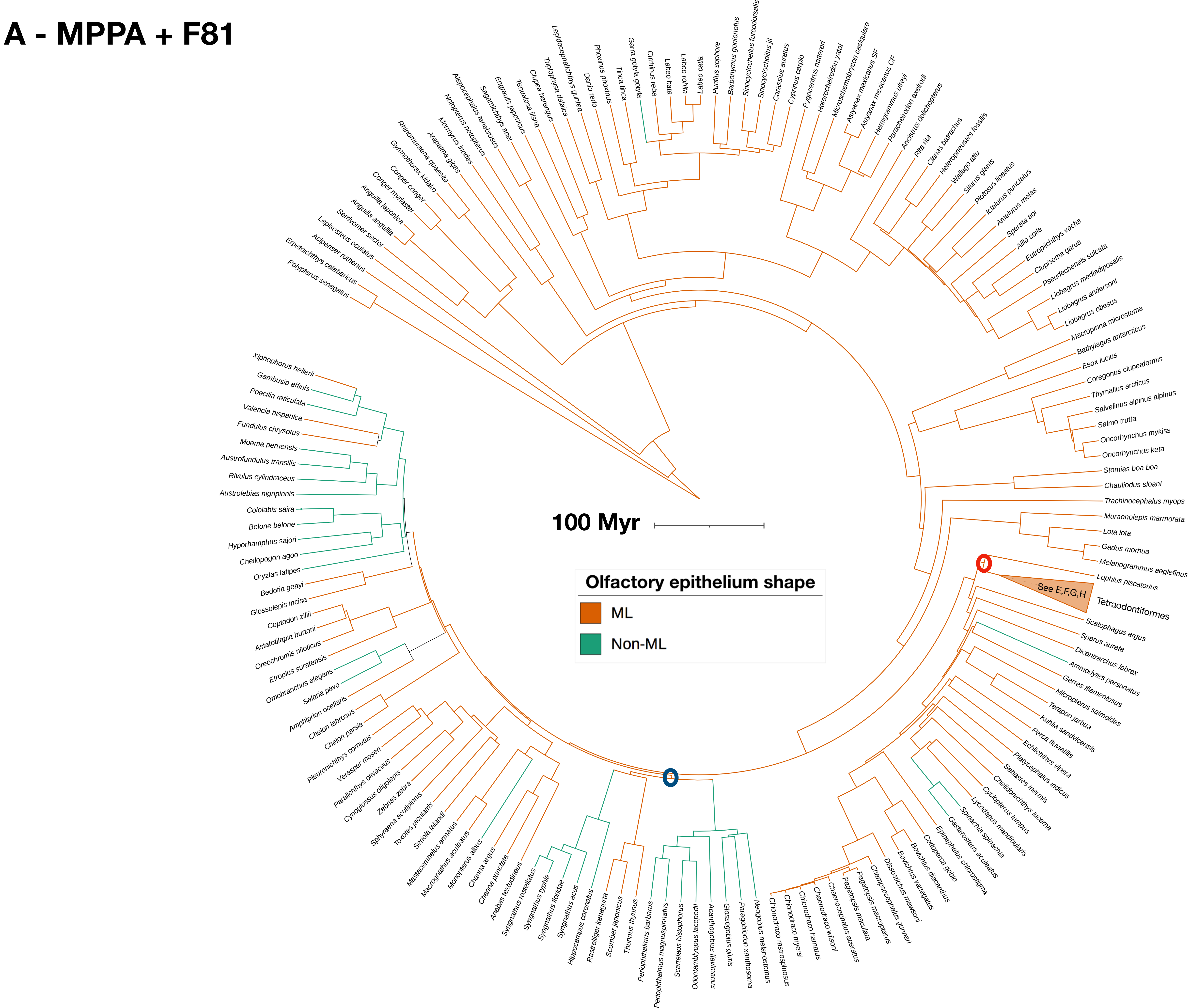
A - MPPA + F81
100 Myr
Olfactory epithelium shape
ML
Non-ML
See E,F,G,H
Tetraodontiformes
Polypterus senegalus
Erpetoichthys calabaricus
Acipenser ruthenus
Lepisosteus oculatus
Serrivomer sector
Anguilla anguilla
Anguilla japonica
Conger myriaster
Conger conger
Gymnothorax kidako
Rhinomuraena quaesita
Arapaima gigas
Mormyrus iriodes
Notopterus notopterus
Alepocephalus tenebrosus
Sagamichthys abei
Engraulis japonicus
Tenualosa ilisha
Clupea harengus
Triplophysa dalaica
Lepidocephalichthys guntea
Danio rerio
Phoxinus phoxinus
Tinca tinca
Garra gotyla gotyla
Cirrhinus reba
Labeo bata
Labeo rohita
Labeo catla
Puntius sophore
Barbonymus gonionotus
Sinocyclocheilus furcodorsalis
Sinocyclocheilus jii
Carassius auratus
Cyprinus carpio
Pygocentrus nattereri
Heterocheirodon yatai
Microschemobrycon casiquiare
Astyanax mexicanus SF
Astyanax mexicanus CF
Hemigrammus ulreyi
Paracheirodon axelrodi
Ancistrus dolichopterus
Rita rita
Clarias batrachus
Heteropneustes fossilis
Wallago attu
Silurus glanis
Plotosus lineatus
Ictalurus punctatus
Ameiurus melas
Sperata aor
Ailia coila
Eutropiichthys vacha
Clupisoma garua
Pseudecheneis sulcata
Liobagrus mediadiposalis
Liobagrus andersoni
Liobagrus obesus
Macropinna microstoma
Bathylagus antarcticus
Esox lucius
Coregonus clupeaformis
Thymallus arcticus
Salvelinus alpinus alpinus
Salmo trutta
Oncorhynchus mykiss
Oncorhynchus keta
Stomias boa boa
Chauliodus sloani
Trachinocephalus myops
Muraenolepis marmorata
Lota lota
Gadus morhua
Melanogrammus aeglefinus
Lophius piscatorius
Scatophagus argus
Sparus aurata
Dicentrarchus labrax
Ammodytes personatus
Gerres filamentosus
Micropterus salmoides
Terapon jarbua
Kuhlia sandvicensis
Perca fluviatilis
Echiichthys vipera
Platycephalus indicus
Sebastes inermis
Chelidonichthys lucerna
Cyclopterus lumpus
Lycodapus mandibularis
Spinachia spinachia
Gasterosteus aculeatus
Epinephelus chlorostigma
Cottoperca gobio
Bovichtus diacanthus
Bovichtus variegatus
Dissostichus mawsoni
Champsocephalus gunnari
Pagetopsis macropterus
Pagetopsis maculata
Chaenocephalus aceratus
Chaenodraco wilsoni
Chionodraco hamatus
Chionodraco myersi
Chionodraco rastrospinosus
Neogobius melanostomus
Paragobiodon xanthosoma
Glossogobius giuris
Acanthogobius flavimanus
Odontamblyopus lacepedii
Scartelaos histophorus
Periophthalmus magnuspinnatus
Periophthalmus barbarus
Thunnus thynnus
Scomber japonicus
Rastrelliger kanagurta
Hippocampus coronatus
Syngnathus acus
Syngnathus floridae
Syngnathus typhle
Syngnathus rostellatus
Anabas testudineus
Channa punctata
Channa argus
Monopterus albus
Macrognathus aculeatus
Mastacembelus armatus
Seriola lalandi
Toxotes jaculatrix
Sphyraena acutipinnis
Zebrias zebra
Cynoglossus oligolepis
Paralichthys olivaceus
Verasper moseri
Pleuronichthys cornutus
Chelon parsia
Chelon labrosus
Amphiprion ocellaris
Salaria pavo
Omobranchus elegans
Etroplus suratensis
Oreochromis niloticus
Astatotilapia burtoni
Coptodon zillii
Glossolepis incisa
Bedotia geayi
Oryzias latipes
Cheilopogon agoo
Hyporhamphus sajori
Belone belone
Cololabis saira
Austrolebias nigripinnis
Rivulus cylindraceus
Austrofundulus transilis
Moema peruensis
Fundulus chrysotus
Valencia hispanica
Poecilia reticulata
Gambusia affinis
Xiphophorus hellerii

Fig. S11

B - DOWNPASS

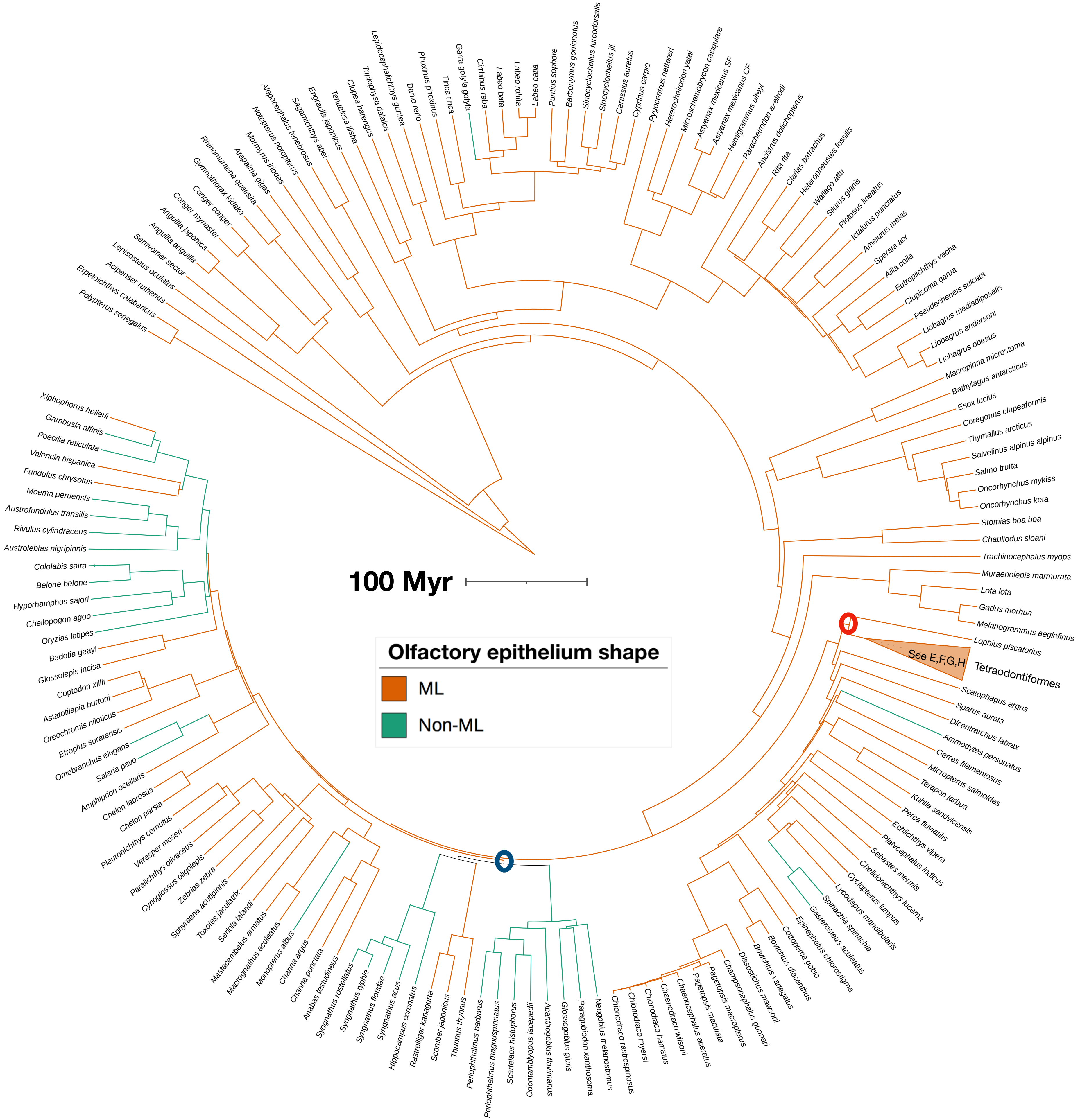

Fig. S11

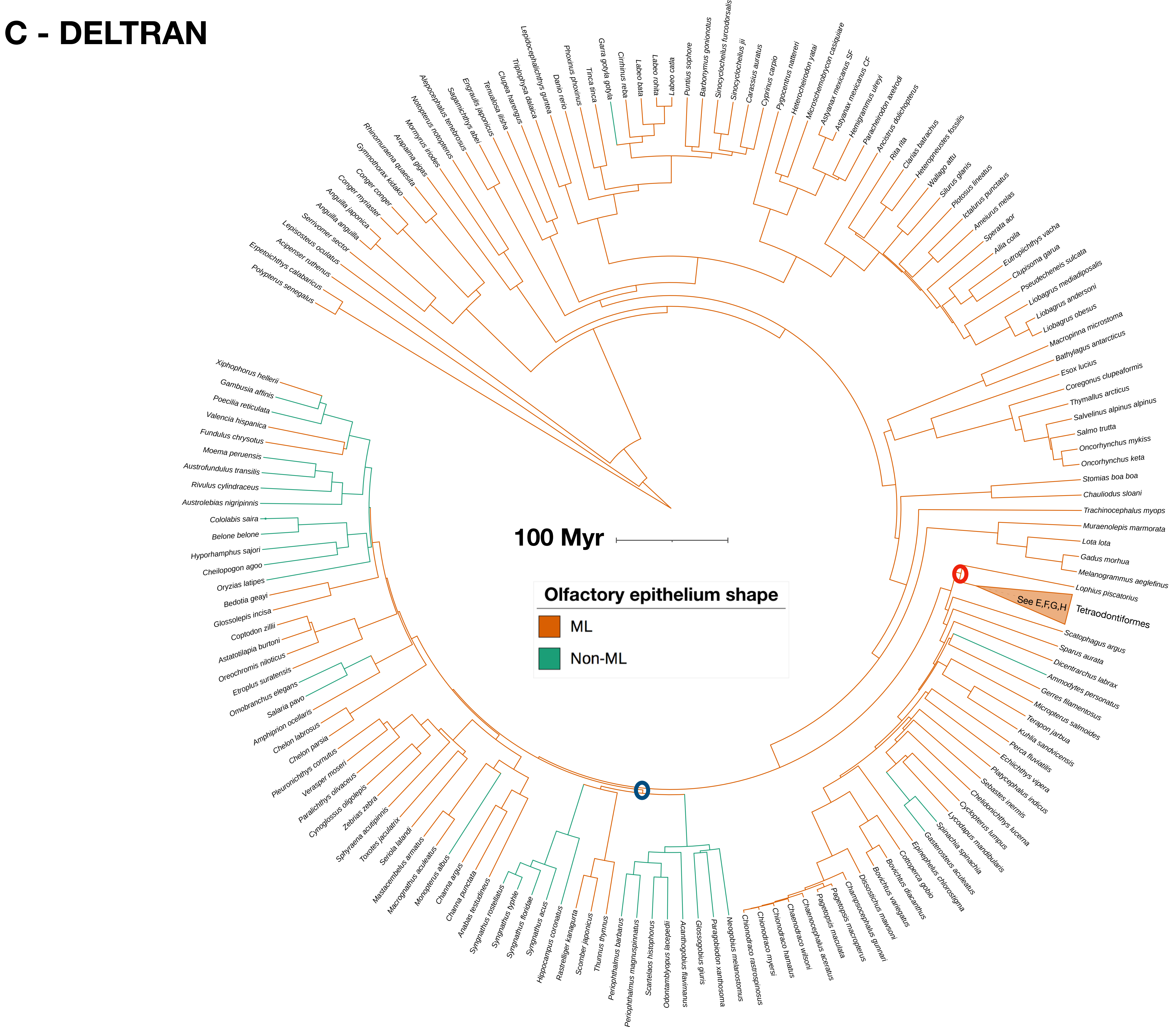

C - DELTRAN
100 Myr
Olfactory epithelium shape
ML
Non-ML
Polypterus senegalus
Erpetoichthys calabaricus
Acipenser ruthenus
Lepisosteus oculatus
Serrivomer sector
Anguilla anguilla
Anguilla japonica
Conger myriaster
Conger conger
Gymnothorax kidako
Rhinomuraena quaesita
Arapaima gigas
Mormyrus iriodes
Notopterus notopterus
Alepocephalus tenebrosus
Sagamichthys abei
Engraulis japonicus
Tenualosa ilisha
Clupea harengus
Triplophysa dalaica
Lepidocephalichthys guntea
Danio rerio
Phoxinus phoxinus
Tinca tinca
Garra gotyla gotyla
Cirrhinus reba
Labeo bata
Labeo rohita
Labeo catla
Puntius sophore
Barbonymus gonionotus
Sinocyclocheilus furcodorsalis
Sinocyclocheilus jii
Carassius auratus
Cyprinus carpio
Pygocentrus nattereri
Heterocheirodon yatai
Microschemobrycon casiquiare
Astyanax mexicanus SF
Astyanax mexicanus CF
Hemigrammus ulreyi
Paracheirodon axelrodi
Ancistrus dolichopterus
Rita rita
Clarias batrachus
Heteropneustes fossilis
Wallago attu
Silurus glanis
Plotosus lineatus
Ictalurus punctatus
Ameiurus melas
Sperata aor
Ailia coila
Eutropiichthys vacha
Clupisoma garua
Pseudecheneis sulcata
Liobagrus mediadiposalis
Liobagrus andersoni
Liobagrus obesus
Macropinna microstoma
Bathylagus antarcticus
Esox lucius
Coregonus clupeaformis
Thymallus arcticus
Salvelinus alpinus alpinus
Salmo trutta
Oncorhynchus mykiss
Oncorhynchus keta
Stomias boa boa
Chauliodus sloani
Trachinocephalus myops
Muraenolepis marmorata
Lota lota
Gadus morhua
Melanogrammus aeglefinus
Lophius piscatorius
See E,F,G,H
Tetraodontiformes
Scatophagus argus
Sparus aurata
Dicentrarchus labrax
Ammodytes personatus
Gerres filamentosus
Micropterus salmoides
Terapon jarbua
Kuhlia sandvicensis
Perca fluviatilis
Echiichthys vipera
Platycephalus indicus
Sebastes inermis
Chelidonichthys lucerna
Cyclopterus lumpus
Lycodapus mandibularis
Spinachia spinachia
Gasterosteus aculeatus
Epinephelus chlorostigma
Cottoperca gobio
Bovichtus diacanthus
Bovichtus variegatus
Dissostichus mawsoni
Champsocephalus gunnari
Pagetopsis macropterus
Pagetopsis maculata
Chaenocephalus aceratus
Chaenodraco wilsoni
Chionodraco hamatus
Chionodraco myersi
Chionodraco rastrospinosus
Neogobius melanostomus
Paragobiodon xanthosoma
Glossogobius giuris
Acanthogobius flavimanus
Odontamblyopus lacepedii
Scartelaos histophorus
Periophthalmus magnuspinnatus
Periophthalmus barbarus
Thunnus thynnus
Scomber japonicus
Rastrelliger kanagurta
Hippocampus coronatus
Syngnathus acus
Syngnathus floridae
Syngnathus typhle
Syngnathus rostellatus
Anabas testudineus
Channa punctata
Channa argus
Monopterus albus
Macrognathus aculeatus
Mastacembelus armatus
Seriola lalandi
Toxotes jaculatrix
Sphyraena acutipinnis
Zebrias zebra
Cynoglossus oligolepis
Paralichthys olivaceus
Verasper moseri
Pleuronichthys cornutus
Chelon parsia
Chelon labrosus
Amphiprion ocellaris
Salaria pavo
Omobranchus elegans
Etroplus suratensis
Oreochromis niloticus
Astatotilapia burtoni
Coptodon zillii
Glossolepis incisa
Bedotia geayi
Oryzias latipes
Cheilopogon agoo
Hyporhamphus sajori
Belone belone
Cololabis saira
Austrolebias nigripinnis
Rivulus cylindraceus
Austrofundulus transilis
Moema peruensis
Fundulus chrysotus
Valencia hispanica
Poecilia reticulata
Gambusia affinis
Xiphophorus hellerii

Fig. S11

D - ACCTRAN

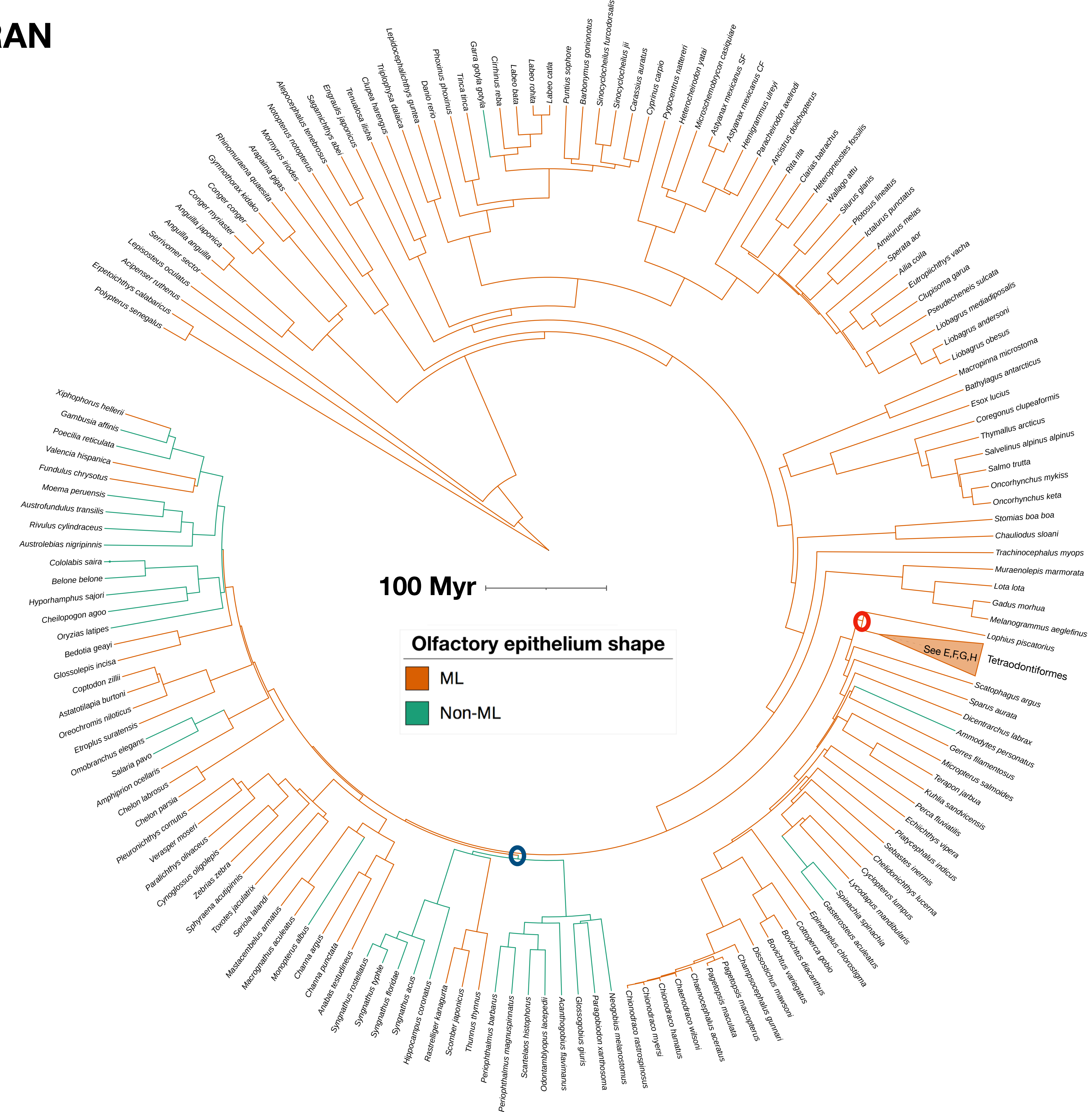

Fig. S11

E - MPPA + F81 , DOWNPASS, DELTRAN, ACCTRAN

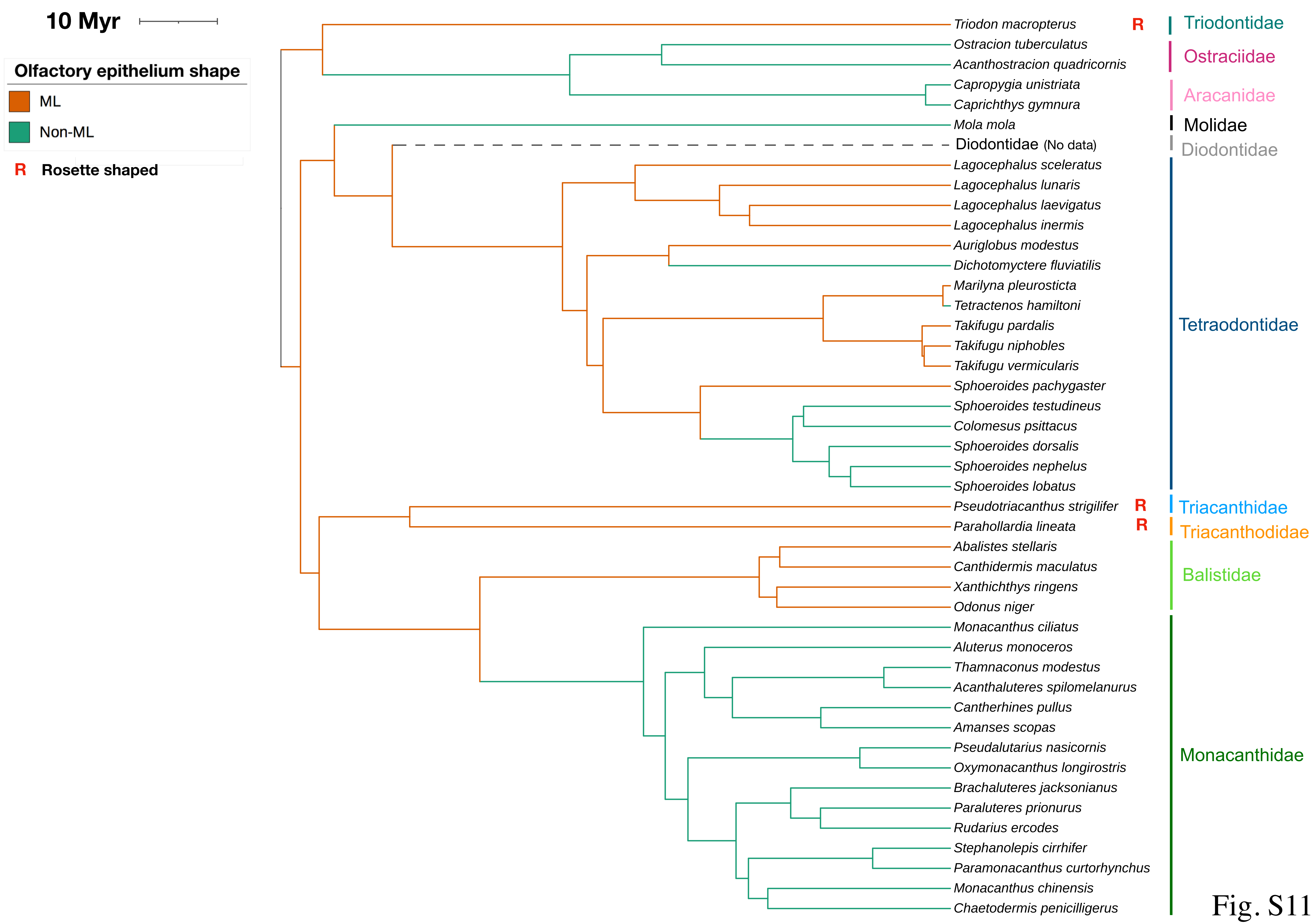

Fig. S11

F - MPPA + F81

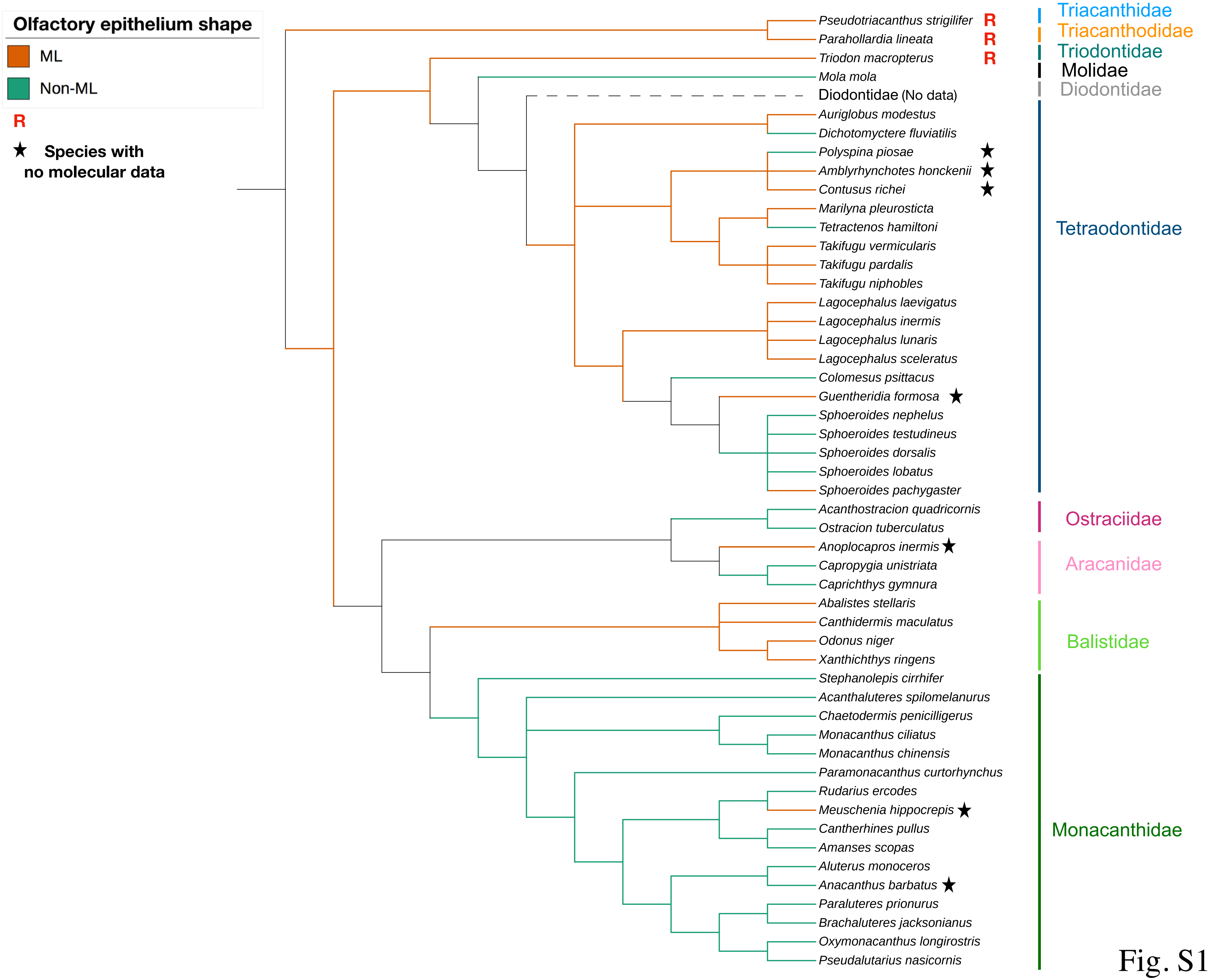

Fig. S11

G - DOWNPASS

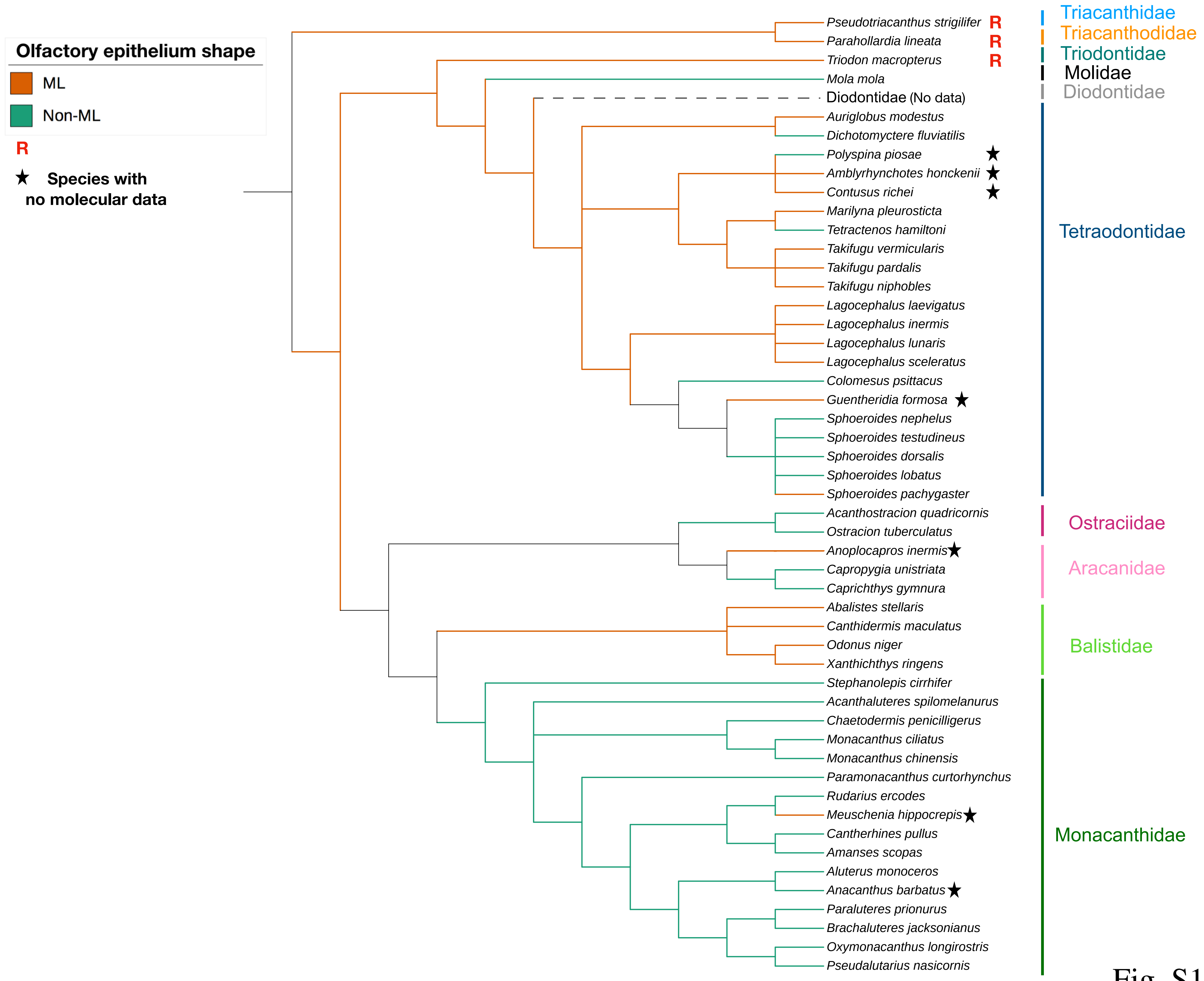

Fig. S11

H - DELTRAN

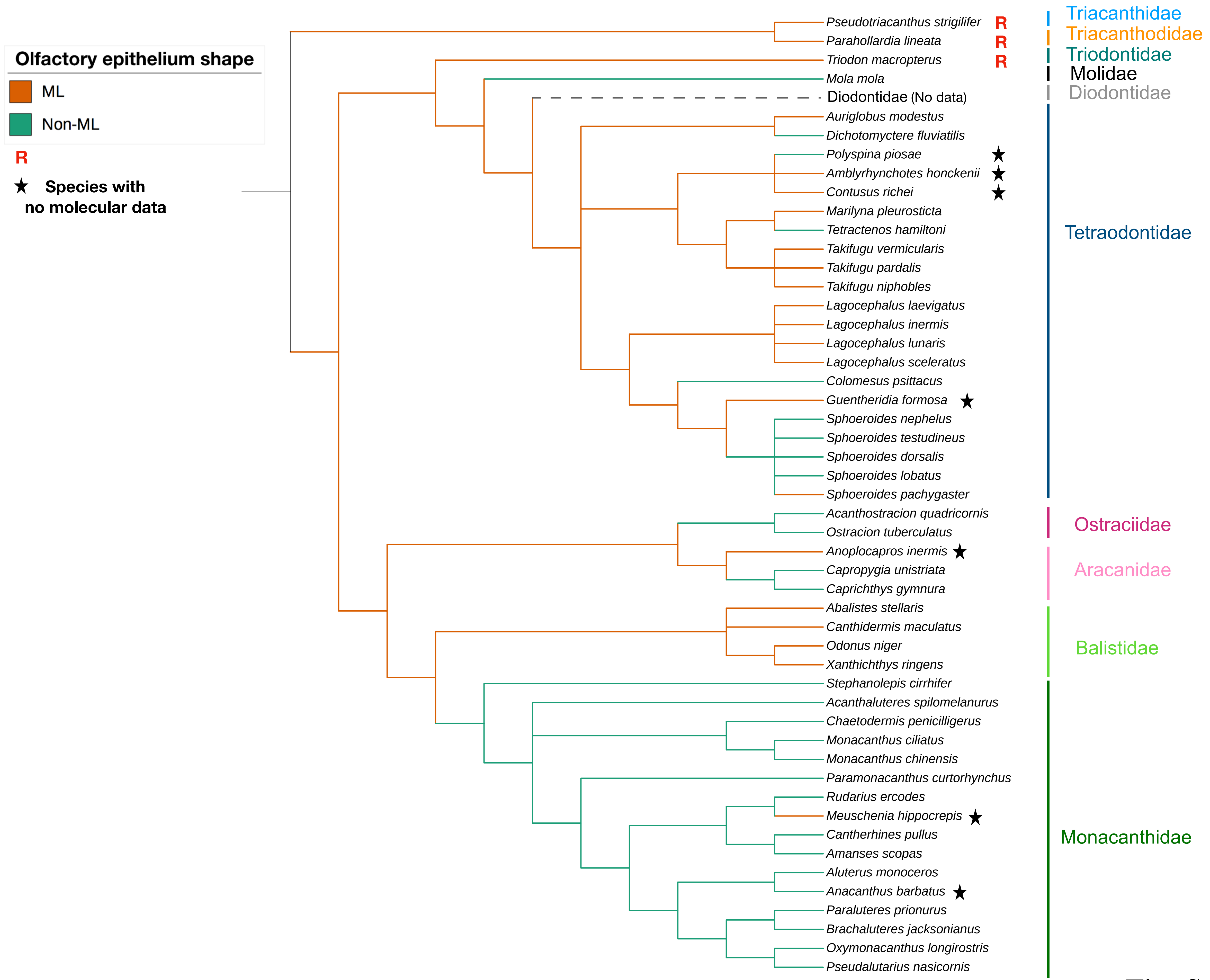

Fig. S11

I - ACCTRAN

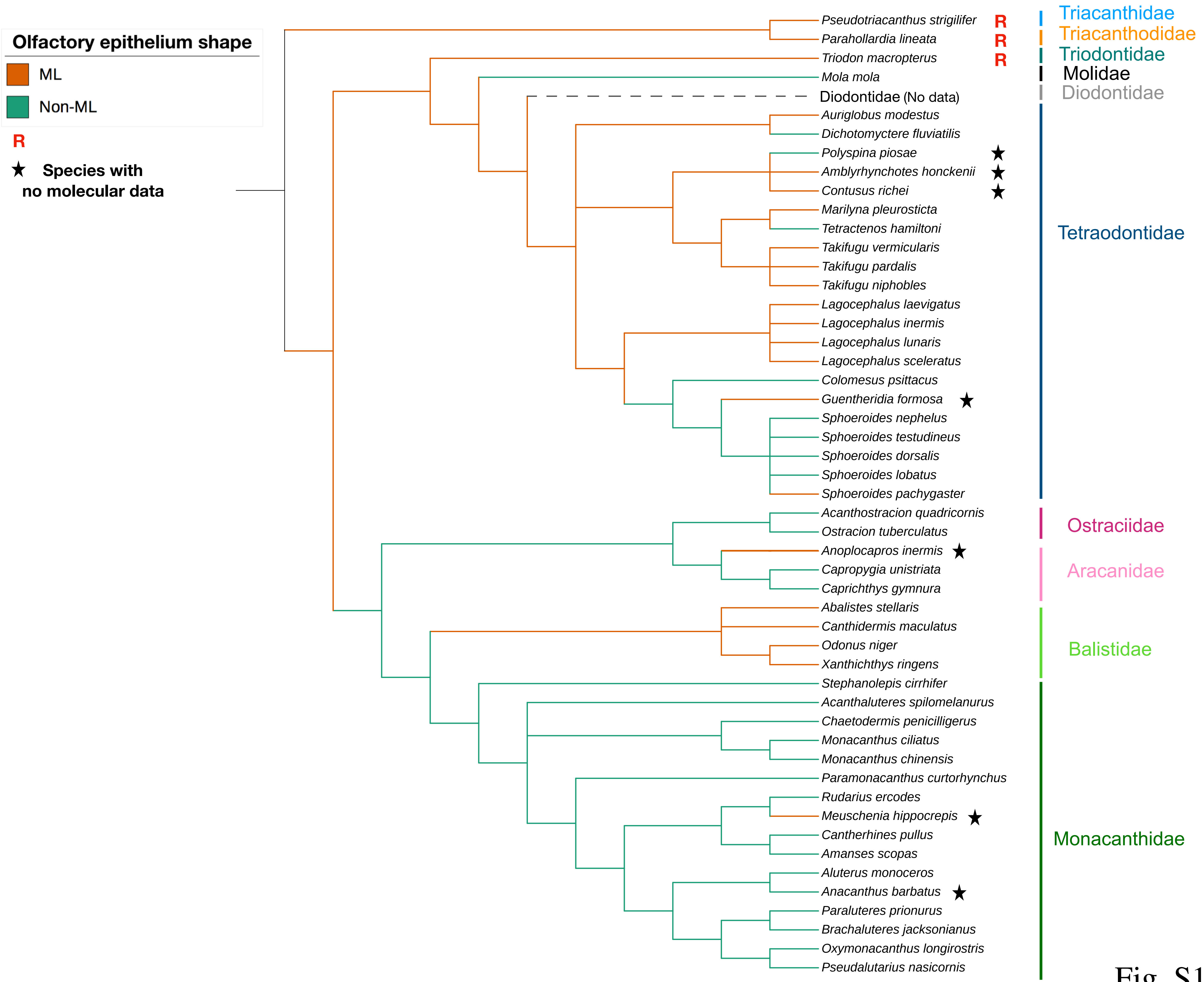

Fig. S11

A

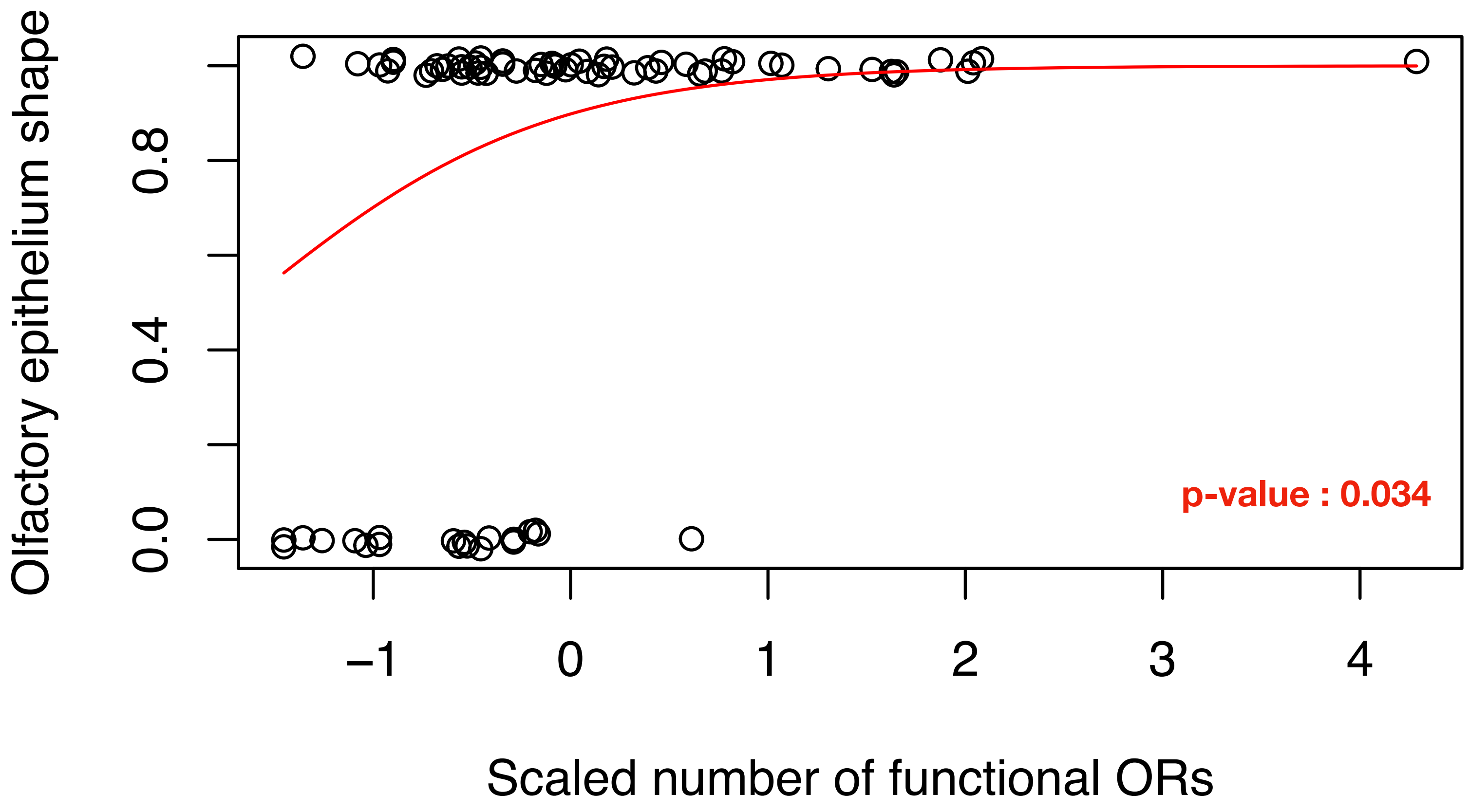

Olfactory epithelium shape
0.0
0.4
0.8
−1
0
1
2
3
4
p-value : 0.034
Scaled number of functional ORs

B

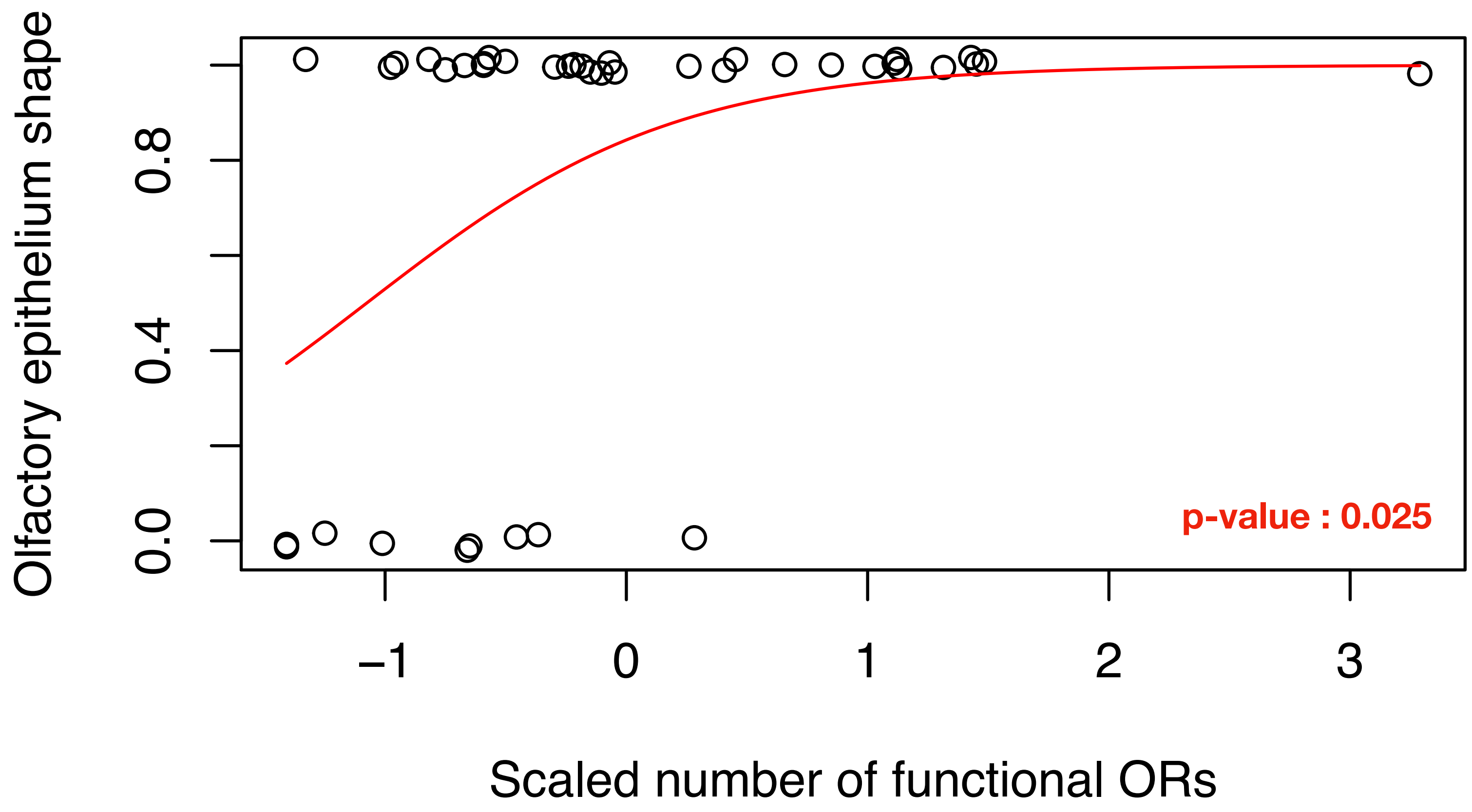

Olfactory epithelium shape
0.0
0.4
0.8
−1
0
1
2
3
p-value : 0.025
Scaled number of functional ORs

Fig. S12

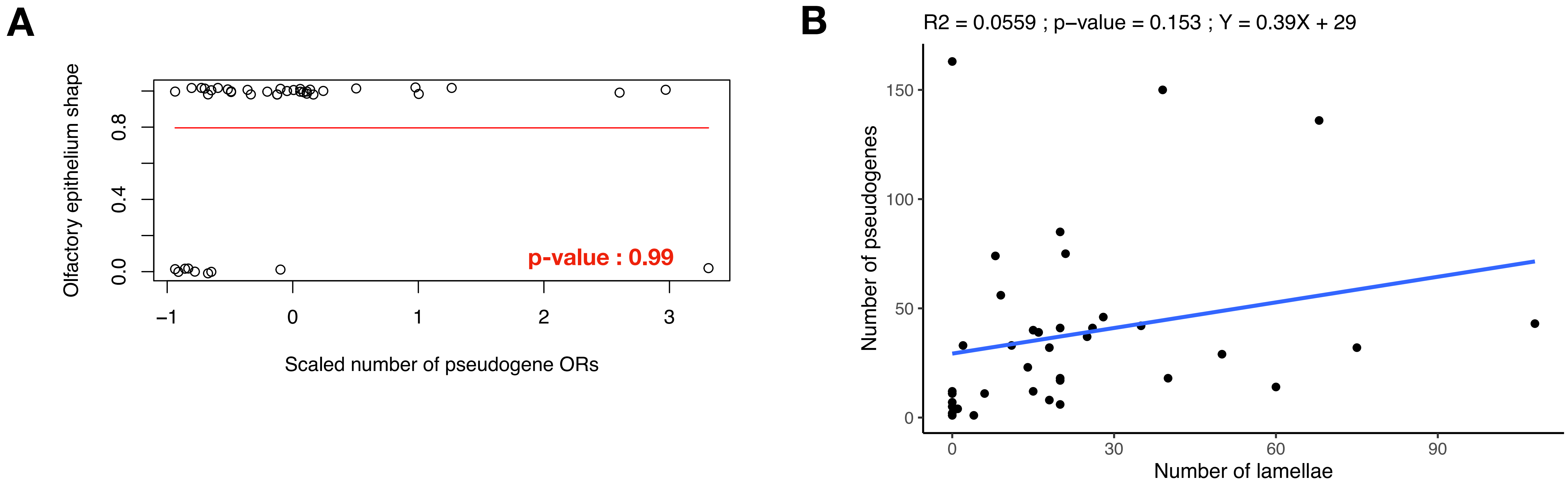
A
Olfactory epithelium shape
0.0
0.4
0.8
p-value : 0.99
−1
0
1
2
3
Scaled number of pseudogene ORs
B
R2 = 0.0559 ; p−value = 0.153 ; Y = 0.39X + 29
Number of pseudogenes
0
50
100
150
0
30
60
90
Number of lamellae

Fig. S13

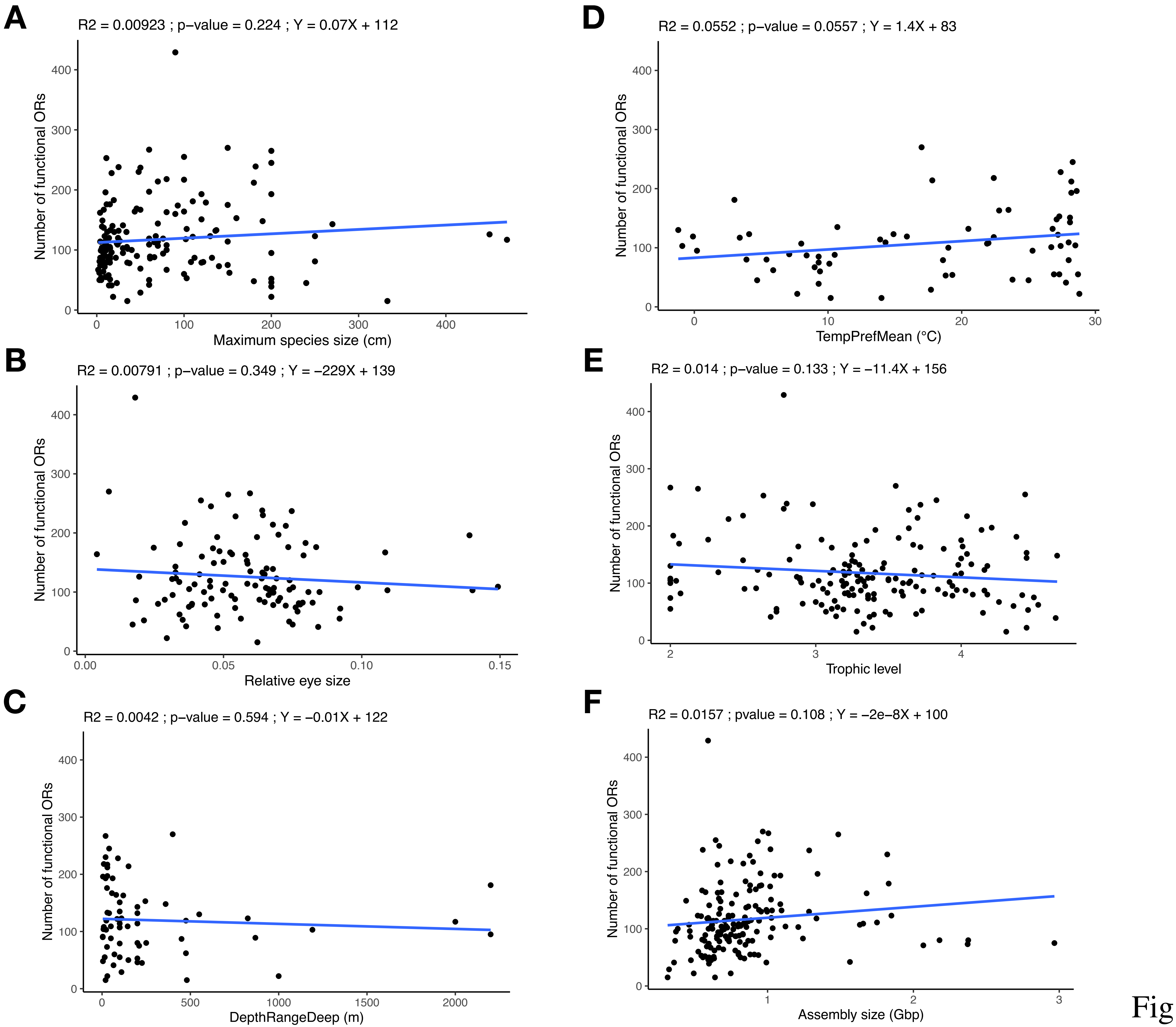
A
R2 = 0.00923 ; p–value = 0.224 ; Y = 0.07X + 112
Number of functional ORs
Maximum species size (cm)
B
R2 = 0.00791 ; p–value = 0.349 ; Y = –229X + 139
Number of functional ORs
Relative eye size
C
R2 = 0.0042 ; p–value = 0.594 ; Y = –0.01X + 122
Number of functional ORs
DepthRangeDeep (m)
D
R2 = 0.0552 ; p–value = 0.0557 ; Y = 1.4X + 83
Number of functional ORs
TempPrefMean (°C)
E
R2 = 0.014 ; p–value = 0.133 ; Y = –11.4X + 156
Number of functional ORs
Trophic level
F
R2 = 0.0157 ; pvalue = 0.108 ; Y = –2e–8X + 100
Number of functional ORs
Assembly size (Gbp)

Fig. S14
